# Excitonic complexes and optical gain in two-dimensional molybdenum ditelluride well below the Mott transition

**DOI:** 10.1038/s41377-020-0278-z

**Published:** 2020-03-10

**Authors:** Zhen Wang, Hao Sun, Qiyao Zhang, Jiabin Feng, Jianxing Zhang, Yongzhuo Li, Cun-Zheng Ning

**Affiliations:** 1 Department of Electronic Engineering, Tsinghua University, 100084 Beijing, China; 2 Frontier Science Center for Quantum Information, 100084 Beijing, China; 3 Beijing National Research Center for Information Science and Technology, 100084 Beijing, China; 4 School of Electrical, Computer, and Energy Engineering, Arizona State University, Tempe, AZ 85287 USA

**Keywords:** Optical physics, Electronics, photonics and device physics

## Abstract

Semiconductors that can provide optical gain at extremely low carrier density levels are critically important for applications such as energy efficient nanolasers. However, all current semiconductor lasers are based on traditional semiconductor materials that require extremely high density levels above the so-called Mott transition to realize optical gain. The new emerging 2D materials provide unprecedented opportunities for studying new excitonic physics and exploring new optical gain mechanisms at much lower density levels due to the strong Coulomb interaction and co-existence and mutual conversion of excitonic complexes. Here, we report a new gain mechanism involving charged excitons or trions in electrically gated 2D molybdenum ditelluride well below the Mott density. Our combined experimental and modelling study not only reveals the complex interplay of excitonic complexes well below the Mott transition but also establishes 2D materials as a new class of gain materials at densities 4–5 orders of magnitude lower than those of conventional semiconductors and provides a foundation for lasing at ultralow injection levels for future energy efficient photonic devices. Additionally, our study could help reconcile recent conflicting results on 2D materials: While 2D material-based lasers have been demonstrated at extremely low densities with spectral features dominated by various excitonic complexes, optical gain was only observed in experiments at densities several orders of magnitude higher, beyond the Mott density. We believe that our results could lead to more systematic studies on the relationship between the mutual conversion of excitonic species and the existence of optical gain well below the Mott transition.

## Trion tricks give optical gain

A new mechanism for amplifying optical signals in two-dimensional semiconductors could be used to develop nano-sized lasers with little input power. When high-energy photons strike a semiconductor they produce excitons – pairs comprising an excited electron and the positively-charged ‘hole’ that it leaves behind, or even trions - states comprising two electrons and one hole, or one electron and two holes. However, scientists have so far limited understanding of the many exotic exciton states that can exist and mutually convert. Cun-Zheng Ning at Tsinghua University in Beijing and co-workers studied ultra-thin single and dual layers of molybdenum ditelluride, and observed a complex interplay between excitons and trions. The trion states enabled the system to show optical gain – a vital property for lasers - despite being orders of magnitude below the so-called Mott transition density, a minimum condition for optical gain in conventional semiconductors.

## Introduction

Dynamical processes of quasiparticles, such as excitons and their various associated complexes (including charged excitons or trions, biexcitons, and other highly correlated objects^1^), in solids are at the core of fundamental condensed matter physics. The evolution of physical states from low to high carrier density involves^2–5^ the insulating exciton gas, Bose–Einstein condensate (BEC)^2^, co-existence of various excitonic complexes, and crossover or transition to conducting electron–hole plasmas (EHPs) or electron–hole liquids (EHLs)^3^ through the Mott transition (MT)^4^. Many fundamental issues associated with the evolution of such quantum quasiparticles and the related phase transitions, especially in the intermediate density regime involving highly correlated complexes, remain to be understood. On the other hand, the mutual conversions of these excitonic complexes and related phase transitions are also profoundly linked to the natures of different optical processes in semiconductors as the carrier density increases^5,6^. The light–semiconductor interaction thus plays important dual roles: As an information reporter, the interaction reveals the intrinsic dynamics of the evolution, co-existence, and mutual conversion of various excitonic complexes in their path towards a degenerate EHP or EHL through the MT, providing a deep understanding of the physical processes when the carrier density is successively increased. At the same time, different conversion processes among various excitonic complexes provide novel absorption or emission mechanisms, forming the physical foundations for photonic functionalities, including lasers, solar cells, light-emitting diodes, and many other devices. While optical processes are well understood in the two extremes, the pure exciton gas and highly degenerate EHP, the intermediate stages involving various excitonic complexes are much less understood. A recent study^7^ showed an interesting relationship between the Mott density (MD) and the transparency density at which optical gain or stimulated emission occurs, especially in low-dimensional systems. Limited study of the stimulated emission through excitonic complexes in the intermediate density regime has produced both scientific discoveries and technological breakthroughs in semiconductor photonics research, such as optical gain based on excitons^8,9^, biexcitons^10,11^, trions^12,13^, and polariton scattering^14^ in III–V or II–VI compound semiconductors or the multiexciton^15^ and single-exciton^16^ gain observed in nanocrystals. These relatively rare gain mechanisms have resulted in lasing demonstrations at much lower pumping thresholds than conventional lasers, which require higher pumping above the MD.

The advent of 2D materials provides a new impetus to study the issues discussed above due to the dramatically weakened screening of the Coulomb interaction. This allows the rich physics related to excitonic complexes, the associated mutual conversions, optical transitions, and phase transitions, such as the MT, and device applications to be explored over a larger range of energies and carrier densities, at higher temperatures, over a larger range of control parameters and with more excitonic species that can co-exist and mutually convert than in conventional III–V or II–VI semiconductors. Among many excitonic complexes are excitons, biexcitons, and trions, an important type of quasiparticle consisting of an exciton and an electron or a hole. While the existence and basic optical features of these excitonic complexes have been investigated^17–23^, the possibility of associated optical gain has not been studied. In related developments, coupling of 2D materials with photonic nano- and microcavities has attracted great interest, leading to demonstrations of 2D material-based stimulated emission and lasing^24–29^. One of the most fundamental questions is the existence and origin or physical mechanism of optical gain, a prerequisite for lasing, in a 2D material. The lasing in these experiments occurs at very low pumping levels of a few W/cm^2 ^^24,25^ or MW/cm^2^^ 26^. The corresponding carrier density levels are approximately 10^6^ or 10^11^ cm^−2^. However, the possibility of optical gain associated with various excitonic complexes below the MT density has not been explored. In several interesting recent developments, optical gain, the MT, and the related giant bandgap renormalization were demonstrated at extremely high density levels on the order of 10^14^ cm^−2^ experimentally^30^ or theoretically^31,32^. The orders of magnitude discrepancy in the carrier densities between the laser demonstrations and the existence of optical gain after the MT raises important questions. For example, does optical gain in 2D materials require them to be in the degenerate EHP state, or can it occur before the MT, supported by excitonic complexes? More importantly, a fundamental question is whether there are more general and profound interrelationships between various excitonic complexes and the stimulated emissions among them in the intermediate density regime. The answers to these questions will provide not only key insights into the co-existence and mutual conversion of various excitonic complexes but also a physical basis for operating 2D material-based photonic devices, such as lasers, at much lower excitation levels and much higher temperatures than in conventional semiconductor lasers.

## Results

In this paper, we study the co-existence of and mutual conversion between excitons and trions in electrically gated monolayer and bilayer molybdenum ditelluride (MoTe_2_) (see the “Methods” section for details). Such electrical gate control was previously used to study trions in a MoS_2_ field-effect transistor^17^, to explore the conversion dynamics between excitons and trions^18–20^, or to study light-emitting diodes^33,34^. We conducted systematic micro-photoluminescence (µ-PL) and reflectance spectroscopy (see the “Methods“ section for details) on electrically gated MoTe_2_ devices using continuous wave (CW) lasers. Our intention was to probe the inner mechanisms of the co-existence of or transition kinetics among various excitonic complexes as the pumping increased and to investigate the possibility, origin, and physical mechanism of optical gain in such low-density regimes.

**Fig. 1:**
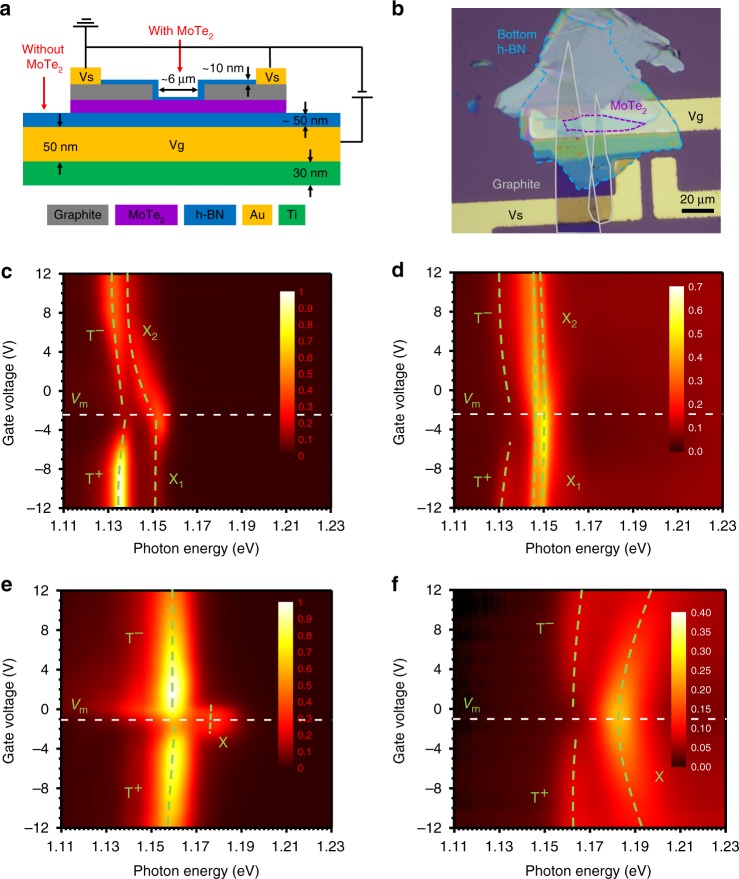
Sample structure and basic optical properties. **a** Schematic of the electrically gated MoTe_2_ device. Monolayer or bilayer MoTe_2_ flakes of side lengths typically over 6 μm were encapsulated with hexagonal boron nitride (h-BN). The entire structure was placed on top of a SiO_2_ (300 nm)/Si substrate covered with Au/Ti electrodes. **b** Optical microscope image of a fabricated device with MoTe_2_ marked by the purple dashed lines. The grey solid line and blue dashed line indicate the regions of graphite contacts and h-BN, respectively. PL **c**, **e** and absorption **d**, **f** maps in the gate voltage and photon energy plane for bilayer **c**, **d** and monolayer **e**, **f** MoTe_2_. Different excitonic species are well resolved, including exciton (X_1_ and X_2_), electron–trion (T^−^) and hole-trion (T^+^) states. The green dashed lines indicate the maxima of the spectral features, while the horizontal white dashed lines indicate the maxima of excitons and minima of trions

Figure 1 shows a schematic diagram (Fig. 1a) and an optical microscope image (Fig. 1b) of the electrically gated sample structure with a bottom Au/Ti electrode (*Vg*) separated by an ~50 nm h-BN film from the 2D MoTe_2_ layer for electrical control of charges. The top contact (*Vs*) consisted of two stripes of graphite separated by ~6 µm to enable laser excitation and collection of the reflectance signal from MoTe_2_. The absorption (*α*(*e*)) and optical gain (*g*(*e*)) spectra at a given pumping level, *e*, are related to the differential reflectance (see Supplementary Information, SI, Section 1 for further discussion) as follows:

1 $$g\left( e \right) = - \alpha (e) \propto \frac{{\left[ {R\left( {e,p,s} \right) - R\left( {0,p,0} \right)} \right] - R\left( {e,0,s} \right)}}{{R\left( {0,p,0} \right)}}$$

where *R*(*e*,*p*,*s*) represents the reflection spectrum in the presence of CW-laser excitation (“*e*”), a broadband probe (“*p*”), and the sample (“*s*”). A zero (“0”) in the respective positions represents the absence of excitation, the probe, or the sample, as indicated in Fig. 1a. It is important that PL (*R*(*e*,0,*s*)) is subtracted in the numerator of Eq. (1), since, by definition, optical gain or absorption is the response of a material to a weak probe. This subtraction is important in CW experiments, as opposed to ultrafast pump-probe measurements, where the PL process has not occurred during the short delay between the excitation and probe pulses. Thus, the intrinsic absorption (*α*(0)) of a material without laser excitation is given by

2 $$- \alpha (0) \propto \frac{{\left[ {R\left( {0,p,s} \right) - R\left( {0,p,0} \right)} \right] - R\left( {0,0,s} \right)}}{{R\left( {0,p,0} \right)}} = \frac{{R\left( {0,p,s} \right) - R\left( {0,p,0} \right)}}{{R\left( {0,p,0} \right)}}$$

Equations (1) and (2) are referred to as the differential reflectance and are commonly used for the determination of the optical gain or absorption of a thin film. As shown in SI Section 2 in more detail, the determination of the optical gain can be complicated due to the effects of substrates with metal.

The PL and absorption (processed using Eq. (2)) spectra are shown in Fig. 1c–f for bilayer MoTe_2_ samples measured at 4 K (Fig. 1c, d) and monolayer MoTe_2_ samples measured at 10 K (Fig. 1e, f) as the gate voltage is varied over ±12 V. Stacked line plots of the same data are reproduced in SI Section 3 (Fig. S6) to more clearly display the spectral features and mutual conversion of excitonic species. We see from both the PL and absorption spectra that the intensity and spectral positions of trions and excitons can be effectively controlled by the gate voltage. While the trion intensity increases with gate voltage, the exciton intensity decreases due to the conversion of excitons into trions with increasing number of electrons or holes. In both the monolayer and bilayer cases, the trion intensities show a minimum at negative voltages, denoted by *V*_m_ (−2.5 V for the bilayer and −1 V for the monolayer), indicating the initial existence of negative charges in the ungated samples. Both the PL and absorption spectra of the monolayer samples show certain symmetric features with respect to *V*_m_, where the exciton feature is the maximum. However, the corresponding spectral features of the bilayer samples show more complicated behaviour. For almost all the bilayer samples measured, there are two neutral excitons (X_1_ and X_2_) in both the PL and absorption spectra that shift and convert with changing gate voltage. Similar two-exciton features in bilayer samples were also previously observed in MoTe_2_^34^. The trion absorption peak is only observable at very high gate voltages. We also noted in Fig. 1c that the emission for hole trions (T^+^) for the bilayer sample is much stronger than that for electron trions (T^−^).

**Fig. 2:**
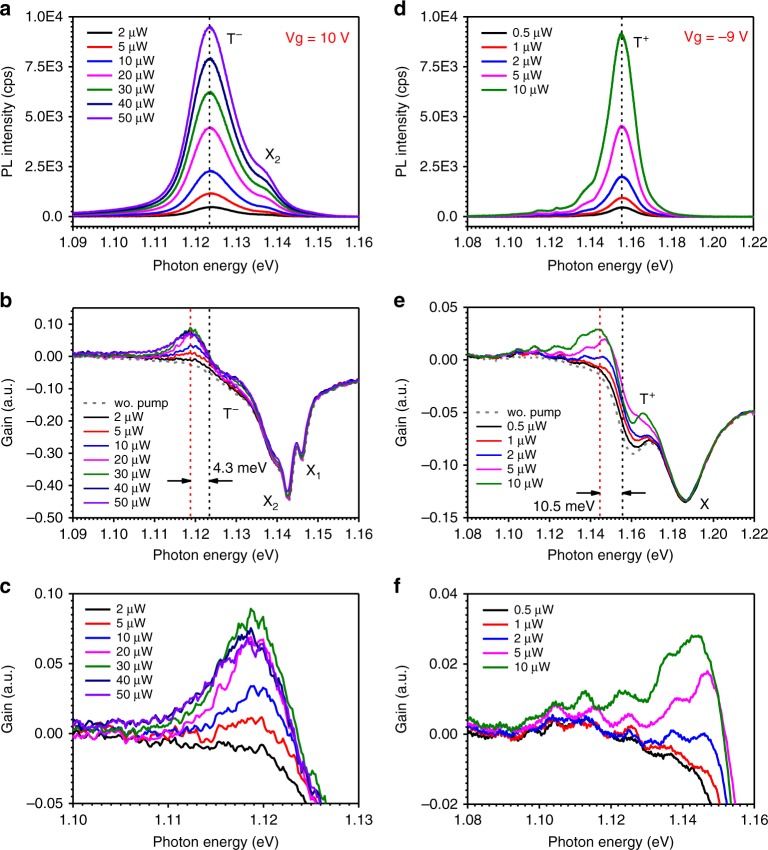
Photoluminescence and optical gain spectra. Pump power-dependent PL **a** and optical gain spectra **b** for the bilayer sample measured at a gate voltage of +10 V. The gain peak is ~4.3 meV below the trion PL peak, as indicated by the two dashed lines. The grey dashed curve is the absorption without pumping. **c** Zoomed-in view of figure **b** to more clearly display the pump dependence and peak positions. **d**–**f** Corresponding PL and gain spectra for the monolayer sample measured at a gate voltage of −9 V. The gain peak is ~10.5 meV below the trion PL peak

Figure 2a shows PL spectra for the same bilayer sample as in Fig. 1c, d as a function of excitation level at 10 V. The PL spectra predominantly show two peaks centred at 1.137 and 1.124 eV, labelled X_2_ and T^−^, which correspond to the exciton and trion for bilayer MoTe_2_, respectively, consistent with previous observations^19,34–37^. Figure 2b shows a series of differential reflectance spectra with increasing excitation, processed using Eq. (1). We see that there is minimum bleaching of the absorption near the two excitons (X_1_ and X_2_) within the relatively weak pump range. However, there is a quite pronounced change in the absorption features at ~1.119 eV; eventually, the absorption is above the background level (see further discussion in SI Section 2, especially Fig. S4 for data processing), or optical gain occurs near 1.119 eV and increases with increasing pumping. A zoomed-in view of the gain spectra is shown in Fig. 2c to clearly display the pump dependence and peak positions of the gain spectra. The optical gain reaches a maximum at a pumping level of 30 µW. It should be noted that the optical gain is different from the signal enhancement, which is a measure of absorption reduction by pumping, defined as the absorption change with and without laser excitation (see SI Section 4 for further discussion).

Figure 2d–f shows the results of similar measurements for the same monolayer sample as in Fig. 1e, f at −9 V. The PL spectra are dominated by hole–trion emission (T^+^), centred at 1.155 eV. The exciton emission is extremely weak and not visible (see also Fig. 1e). Optical gain occurs near 1.145 eV. The appearance of gain with increasing pumping and the trend of the change are very similar to those for the bilayer case. There is a general trend for both bilayer and monolayer samples that can be described as follows: with increasing pumping, the absorption features near the trion energy start to saturate (see Fig. 2b, e), and a peak emerges slightly below the trion emission energy above the background, indicating the appearance of optical gain. For a given gate voltage, the gain peak saturates at a sufficiently high pumping level (e.g., 30 µW in Fig. 2c). However, the features of the gain spectra become visibly more irregular for the monolayer sample due to the existence of defect states below the trion peak, which are clearly visible in the PL spectra (see defect features in Fig. 2d below 1.14 eV). We note throughout this research that the gain spectrum becomes significantly noisy whenever defects become visible in the PL spectrum. This is especially the case for monolayer samples with significant defect emission. The noisy features in the gain spectrum (e.g., in Fig. 2f) are mainly the results of error amplification in the data processing to obtain the differential reflectance using Eq. (1). Figure S8 in SI Section 5 shows the respective integrated PL intensities of excitons and trions as a function of pumping, showing a nearly linear dependence. Such linear dependence excludes exciton–exciton scattering^8^ or biexcitons^10,11^ as the gain mechanism, since both feature a quadratic dependence of the PL on pumping. Within the range of pumping, no biexciton emission is observed. Thus, the appearance of optical gain, which is spectrally associated with the trion state, likely originates from trions. For a detailed discussion about trion dispersion and the varieties of trions in MoTe_2_, please see SI Section 6 for more details.

**Fig. 3:**
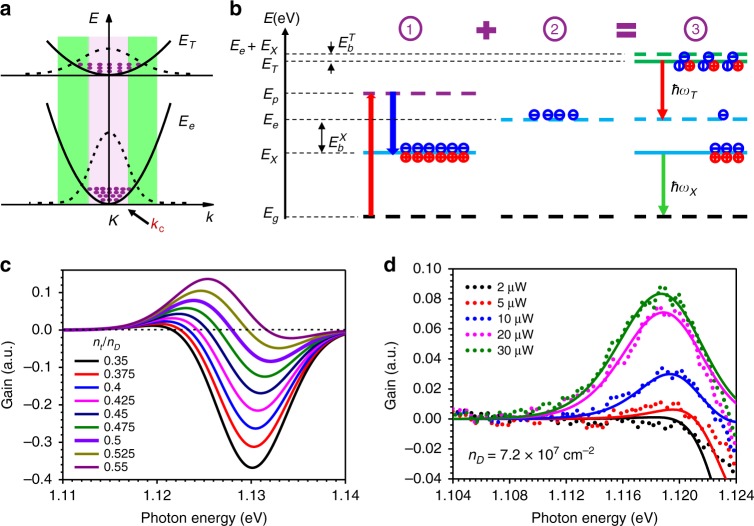
Physical mechanisms and theoretical model for trionic gain. **a** Parabolic bands (solid lines) and electron distributions (dashed lines) in the trion band (*E*_T_) and electron band (*E*_e_). The pink inner band indicates the region around *K* where the absorption process is dominant, while in the outer green bands (separated at *k* = *k*_c_), local population inversion can occur. **b** Schematic of the three key steps of trion formation through exciton generation (*E*_X_) via optical pumping (*E*_P_) from the ground state (*E*_g_) (step 1); pre-existence of electrons (*E*_e_) due to gating or doping (step 2); and possible population distribution among three states (trion, electron, and exciton) and occurrence of population inversion (step 3). *E*_b_^T^ and *E*_b_^X^ denote the binding energies for trions and excitons, respectively. **c** Theoretical absorption and gain spectra at different *n*_t_/*n*_D_ ratios (*n*_t_: trion density; *n*_D_: doping level) from the model (Eq. (3)). **d** Fitting result (solid lines) of the measured gain spectra (dotted lines) from Fig. 2c for the “four-band” model using Eq. (3)

Typically, optical gain appears on the low-energy side of the PL peak and is ~ 4.3 and ~10.5 meV below the trion peak for the bilayer and monolayer samples, respectively. This behaviour and the relative spectral features strongly suggest that the optical gain originates from trions. The physical mechanism of trionic gain was first studied in doped ZnSe quantum wells^12^. According to this understanding, the trion system can be described by a “two-band” model: a ground state of a doped (e.g., n-type) material in which the conduction band, *E*_e_, is filled with a given number of electrons (which could be pre-doped due to defects, gate generated or intentionally doped) and an upper trion band, *E*_T_, which has a much heavier effective mass (*m*_T_ = 2*m*_e_ + *m*_h_ for electron trions), as illustrated in Fig. 3a. The trion formation is more accurately described by the “four-band” model, as illustrated in Fig. 3b, which consists of three key steps: Excitons (bound electron*–*hole pairs) generated through optical pumping (step 1) quickly find their charged partners generated through gating (step 2) to form trions (step 3) by releasing a binding energy of *E*_b_^T^. For each pump-generated exciton, one electron in the lower band is consumed to form a trion in the upper band. The net effect of a pumping photon (at energy *E*_P_) is to decrease (increase) the population of the lower band, *E*_e_ (upper band, *E*_T_), by one. Such a three-step process can lead to population inversion between the trion and electron bands and achieve optical gain, as shown in step 3 of Fig. 3b. The upper limit of the optical gain is reached when all pre-existing electrons form trions with their exciton partners. The maximum gain is limited by the total number of pre-doped electrons (*n*_D_). More precisely, the occupation of the trion state (step 3) at a given pumping level (*n*_p_) is determined by the relative distribution of trions, electrons, and excitons (at *E*_X_). Thus, the co-existence, mutual conversion, and resulting steady-state distribution of free electrons, holes, and all excitonic complexes determine the population inversion and the amount of achievable optical gain.

Interestingly, to achieve positive optical gain, there is no need for the total number of carriers in the trion band to exceed the number in the conduction band, so-called global population inversion. As seen in Fig. 3a, due to the much heavier effective mass in the trion state, the same number of carriers in the trion bands occupies a much larger range in *k*-space, leading to the existence of a crossover *k* point, *k*_c_, such that there is local population inversion for *K−k*_c_ > *k* > *K* + *k*_c_. This leads to a situation where the optical gain provided in the green bands could exceed the absorption in the pink band before global inversion is reached, depending on the pumping and linewidth broadening for a fixed level of doping density. Such an occurrence of optical gain through local population inversion without global inversion was first observed in quantum cascade lasers^38^. However, here, the effect is more pronounced due to the much larger difference in effective masses. More quantitatively, the optical gain is modelled by

3 $$G(\omega ) \propto \mathop {\sum}\limits_k {\left| {\left. {\mu _k} \right|} \right.^2L\left( {k,\omega } \right)} \left( {f_{\mathrm {t}} - f_{\mathrm {e}}} \right)$$

where *f*_t_ and *f*_e_ represent the Fermi distributions of electrons in the trion band and conduction band, respectively. $\left| {\left. {\mu _k} \right|} \right.^2$ is the optical dipole matrix element, and *L*(*k*, *ω*) is a lineshape function (see SI Section 7 for details). The calculated gain spectra for a sequence of increasing ratio, *n*_t_/*n*_D_, are shown in Fig. 3c, where we see that the optical gain appears on the slightly red side of the trion peak energy, consistent with the experimental results. For a fixed electron doping density *n*_D_, the critical condition for population inversion is *n*_t_ = *n*_e_ = 0.5*n*_D_ for a typical two-band system. Since *n*_D_ can be of any low value, in principle, optical gain can occur at an extremely low carrier density. This is in strong contrast to the occurrence of optical gain based on an electron–hole plasma, which requires a very high transparency density. In addition, due to the possibility of local *k*-space population inversion before global inversion, optical gain occurs at a trion density slightly <0.5*n*_D_, as seen in Fig. 3c.

To more quantitatively understand our experimental measurement of the gain spectrum in terms of the “four-band” model and the gain spectra calculated from Eq. (3), we need to determine the steady-state distributions of electrons, excitons, and trions using the well-known mass-action law^3,39^. For electron trions formed by$X + e \leftrightarrow T^ -$, we have $n_{\mathrm {x}}n_{\mathrm {e}} = n_{\mathrm {t}}K\left( T \right)$, where *K*(*T*) is a temperature-dependent equilibrium constant (see SI Section 8 for details). From charge conservation, the trion density *n*_t_ can be calculated as a function of *n*_p_ and *n*_D_. The pumping density, *n*_p_, is determined by the measured absorption coefficient at the pumping wavelength. The doping density, *n*_D_, is determined by best-fitting experimentally measured spectra using Eq. (3) and the calculated trion density (see SI Section 8). The experimental spectra agree with the modelled spectra quite nicely with only a single *n*_D_ (see Fig. 3d). It is important to emphasize that the optical gain occurs below a 5 µW pump power, corresponding to a pumping density of ~3.6 × 10^7^ cm^−2^. The electron doping density obtained by best fitting is ~7.2 × 10^7^ cm^−2^, in good agreement with the “two-band” gain model discussed above in Fig. 3a, with all the populations determined by the “four-band” model in Fig. 3b (step 3). We note that electron–hole plasma gain in 2D materials typically appears at densities on the order of ~10^13^−10^14^ cm^−2^ ^30–32^, or several orders of magnitude higher than that required for the trion gain studied in this paper. The very low density level also excludes exciton-polarons^40,41^ as a possible gain mechanism, which occurs in the presence of a highly degenerate electron gas with Fermi energy close to the trion-binding energy. As shown in Fig. S10 in SI section S7, the electron chemical potential is still negative in our cases, and we are still in the low density regime where the coupling of electrons with excitons mostly displays the character of three-particle trions, not the full many-body version of exciton-polarons.

**Fig. 4:**
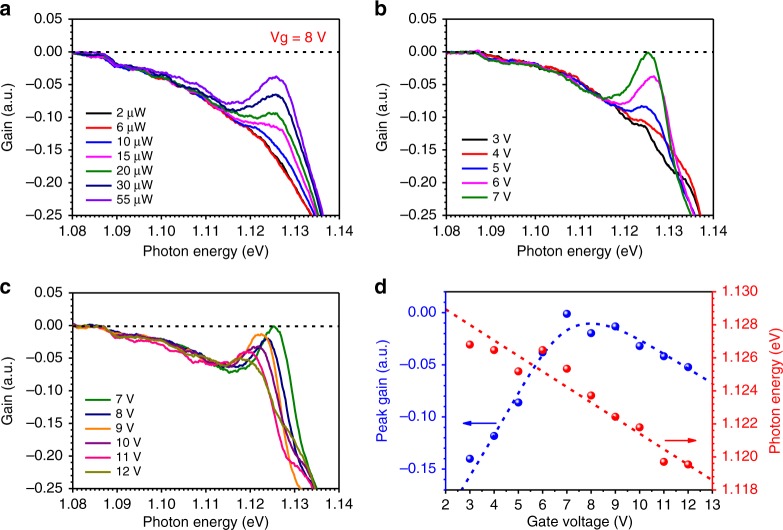
Optical gain of another bilayer sample. **a** Optical gain spectra at several pumping levels for another bilayer sample measured at 4 K and a gate voltage of 8 V. **b**, **c** Optical gain spectra at several gate voltages at 40 µW pumping. The gain spectra are presented in two groups in figures **b**, **c**, separately showing increasing **b** and decreasing **c** trends with the gate voltage. **d** Extracted peak gain values and photon energies of the gain peaks as a function of gate voltage

Figure 4 presents similar gain measurement results for another bilayer MoTe_2_ sample, where both the pumping-dependent gain spectra at a fixed gate voltage (Fig. 4a) and the gain spectra at varying gate voltage for a fixed pumping level (Fig. 4b–d) are shown. Different from the gain features in Fig. 2, the gain spectra in Fig. 4a–c show a tilted negative background at the low photon energy side below 1.115 eV, indicating strong absorption, likely due to the existence of defects. The results are discussed in more detail in SI Section 9. However, despite the negative background, the gain due to trion is clearly visible as a peak that starts to build at ~1.125 eV. As shown in Fig. 4a, optical gain due to trions occurs at ~10 µW pumping (*n*_p_ ~ 7.2 × 10^7^ cm^−2^). A clear pumping-dependent increase in the optical gain is seen at a relatively large gate voltage of 8 V, where a sufficiently high electron density (*n*_D_) is produced by gating. With increasing pumping, an increasing number of excitons are generated that form negative trions with their partner electrons. This translates into a higher population in the trion band, leading to a monotonous increase in the optical gain. The gain evolution for various gate voltages at a fixed pumping level of 40 µW in Fig. 4b, c is especially interesting. The optical gain initially increases with the gate voltage from 3 to 7 V. The 40 µW excitation level produced enough excitons for gate-generated electrons to form trions up to 7 V. Thus, with each increase in the gate voltage, there is an increase in the population in the trion band, leading to an increase in the optical gain in Fig. 4b. The gain starts to decrease when the gate voltage is further increased beyond 8 V, as shown in Fig. 4c. This is because too many electrons are produced at higher gate voltages relative to the number of excitons produced at 40 µW. The trion population in the upper band is capped by the given total exciton number generated by pumping, while the electron population in the lower band keeps increasing with increasing gate voltage beyond 8 V. Such a decrease in the population difference (compared to Fig. 3b, step 3) due to the increased lower band population leads to a continuous decrease in the optical gain as the gate voltage is further increased. This feature is more clearly displayed in Fig. 4d, where the extracted peak gain values and the photon energies of the corresponding gain peaks are plotted versus the gate voltage. The overall negative gain values are due to the coupling to absorption for both defects and excitons. This is discussed in more detail in SI Section 9. The trionic gain model and physical picture presented in Fig. 3 can explain the data here quite satisfactorily. More results at different gate voltages or for other devices are shown in SI Section 10 to provide more evidence of optical gain.

## Discussion

In summary, we have conducted systematic PL and reflectance spectroscopy experiments on electrically gated MoTe_2_ devices to investigate possible optical gain at low excitation levels. Our results on both monolayer and bilayer samples show the clear existence of optical gain at such low carrier density levels, orders of magnitude below the MD. After examining the signatures of optical gain and their relationship with excitons and trions, we could clearly identify the origin of optical gain as being trionic in nature. The physical model and understanding can explain our experimental results quite well. Our results have clearly established the existence and trionic origin of the optical gain measured in 2D MoTe_2_. We emphasize that the optical gain observed well below the MD is of special interest in 2D materials. The main results and key conclusions of this paper are of great importance on several levels. First, while the existence of optical gain and the stimulated emission associated with excitonic complexes in conventional semiconductors have been occasionally studied and are of great importance both in basic condensed matter physics and in possible device applications, a similar study has not yet been carried out for 2D materials. Our results significantly contribute to the understanding of the nature of the co-existence and mutual conversion of various excitonic complexes well below the MD. Second, the existence of optical gain at extremely low density levels is of tremendous importance for fabricating nanolasers with very low threshold and power consumption. As we noted above, the existence of optical gain does not depend on the total number of injected carriers but on the population difference between the trion band and electron band. Thus, the trion gain can in principle exist at any arbitrarily low carrier density level. This possibility opens a wide vista for fabricating extremely low-threshold lasers down to the single charge level^13^.

Importantly, there seems to be a more intricate link between the existence of optical gain and the MT. A recent study^7^ revealed an even closer relationship between the “transparency” density (*N*_t_) and MD (*N*_m_), especially in low-dimensional material systems. In most conventional III–V or II–VI semiconductors, no optical gain typically exists in the low-density regime until a critical or “transparency” density is reached, and optical gain subsequently appears when the carrier density exceeds *N*_t_. Due to the small exciton binding energy in conventional semiconductors, we typically have *N*_t_ > *N*_m_. Almost all the available lasers operate based on this type of optical gain related to the degenerate EHP. *N*_t_ is typically on the order of 10^11^ to 10^12^ cm^−2^ depending on the temperature, requiring high excitation or energy input. Thus, it would be highly desirable to search for the possible existence of optical gain in the low-density regime, well below the MD, e.g., *N* ≪ *N*_m_ < *N*_t_. Such gain mechanisms would be extremely important for lower power, energy efficient device applications, given the current research interests in femto- or attojoule optoelectronics^42^. Excitonic-related gain mechanisms for *N* < *N*_m_ have only been demonstrated in rare cases for conventional semiconductors, including exciton–exciton scattering^8^, localized excitons^9^, localized biexcitons^10^, (free) biexcitons^11^, trions^12,13^, and exciton–polariton scattering^14^. This is why such lasers have not been widely used or manufactured. It is expected that all these rich varieties of gain mechanisms are now observable in 2D materials at room temperature and could find practical use in 2D material-based lasers. At the same time, it is hoped that such a study could reveal more profound links between various gain mechanisms and the co-existence or mutual conversion of various excitonic complexes in the vast range of the low-to-intermediate-density regime below the MD.

As optical gain materials, 2D materials are particularly appealing for several reasons: First, the atomic-level thickness provides the thinnest optical gain materials to potentially achieve the smallest-volume low power lasers, since the total power consumption of a nanolaser is determined by the total volume of the gain material^42^. Second, the ultimate flexibility of 2D materials allows integration with strongly mismatched materials, such as silicon^25^, to realize integrated photonics that do not suffer from mechanical or thermal damage, as in the case of III–V/Si integration. Such heterogeneous Si-2D material integration could be potentially superior to that of traditional III–V semiconductors, which are currently the prevailing choices for silicon photonics but face many challenges due to the mechanical rigidity and small tolerance to lattice mismatches. Finally, the possibility of staggering different 2D materials to form artificial heterostructures “by design” could be extremely important for eventually achieving 2D material-based nanolasers under electrical injection, which represents a remaining challenge. In this regard, the discovery of the trionic optical gain at extremely low carrier density levels is of special importance. Despite extensive efforts, efficient light emission through electrical injection based on 2D materials is still very challenging. One of the key challenges is the poor injection efficiency of carriers into 2D materials and poor carrier confinement, requiring the achievement of an extremely high density (~10^13^–10^14^ cm^−2^) of carriers to achieve optical gain in the electron–hole plasma. Achieving such a high density level through electrical injection is impractical or even impossible for traditional, perfectly fabricated double heterostructures based on III–V and II–VI semiconductors. Our results on trion gain point to an important new possibility of achieving optical gain at densities several orders of magnitude lower, well within the levels achievable using current approaches, such as the use of gate-controlled heterostructures based on 2D materials. Currently, we are actively pursuing such electrical-injection luminescence^43^ as well as coupling of such trion emission into cavity modes^44^.

## Materials and methods

### Fabrication of electrically gated MoTe_2_ devices

Monolayer and bilayer MoTe_2_ were prepared by mechanical exfoliation from commercial bulk crystals (2D semiconductors Inc. and HQ graphene Inc.) and transferred onto a thick polydimethylsiloxane (PDMS) film using the standard dry transfer method. The layer thickness was identified using atomic force microscopy (AFM) and the optical microscope contrast. Prior to any processes, the best-performing MoTe_2_ material was preselected based on photoluminescence (PL) measurement results with a narrow linewidth and a high emission intensity. Metal electrode contacts were predefined on a SiO_2_ (300 nm)/p-Si substrate using photolithography. Au/Ti (50/30 nm) electrodes were then deposited via electron beam evaporation as contacts. After fabrication of the electrodes, a freshly exfoliated hexagonal boron nitride (h-BN) film (~50 nm) was transferred onto one of the predefined Au/Ti electrodes as a dielectric layer. Subsequently, exfoliated MoTe_2_ film was transferred on top of the h-BN film by employing a nanopositioner for precise alignment. Two graphite stripes (~10 nm) were then transferred as top contacts bridging MoTe_2_ and the other Au/Ti electrode. This Au/Ti electrode was grounded, and the other electrode functioned as a back gate, providing electrostatic carriers to the material. Finally, a second thin h-BN (~10 nm) film was transferred on top of MoTe_2_ for protection from contamination. All the transfer processes were carried out with the aid of PDMS as the carrier stamp at moderate heating temperatures to avoid possible sample degradation.

### Optical and electrical characterization system

The optical properties of monolayer and bilayer MoTe_2_ were characterized in a home-built micro-PL system at temperatures of 4 and 10 K, respectively. The sample was attached to the cold finger of a liquid helium cryostat and placed under vacuum. A 632.8 nm (1.96 eV) continuous-wave HeNe laser was used as the pumping source to excite the sample through a ×100 NIR-optimized objective with NA = 0.7. The reflected signal was collected by the same objective and delivered to a grating spectrometer (Princeton Instruments Acton 2560i) equipped with a LN-cooled InGaAs CCD for detection. The pump laser spot size was estimated to be ~4 µm in diameter using the knife-edge method, which is much smaller than the typical size of exfoliated MoTe_2_. The excitation laser power was kept below 100 μW. Power-dependent PL measurements were conducted to determine the exciton/trion peak positions and binding energies of MoTe_2_. For reflectance measurements, a stabilized tungsten halogen lamp (Thorlabs SLS201L) was used as the broadband radiation source with a spot size smaller than that of the pump laser. The reflectance spectra were smoothed by averaging 5–15 adjacent data points, corresponding to an energy range of 0.5–3 meV. The range was tested to ensure that artificial changes to the measured spectra were not induced. Electrically gated PL and absorption measurements were conducted by incorporating a commercial semiconductor parameter analyser (Keysight B1500A). The gate voltage was varied between −12 and +12 V with a step of 0.5 V.

## Supplementary information

SI text+figure

SI figure

## Data Availability

The data that support the findings of this study are available from the corresponding author upon request. Supplementary information accompanies the manuscript on the Light: Science & Applications website (http://www.nature.com/lsa/).

## Code availability

The full code that supports the findings of this study is available from the corresponding author upon request.
