# Supplementary material for: Excitonic complexes and optical gain in two-dimensional molybdenum ditelluride well below the Mott transition
Source: Light Sci Appl. 2020 Mar 10;9:39. doi: 10.1038/s41377-020-0278-z (PMC7064520; doi:10.1038/s41377-020-0278-z)
Supplement: Supplementary file 1 — SI text+figure [file 41377_2020_278_MOESM1_ESM.docx]

**Supplementary Information for**

**Excitonic Complexes and Optical Gain in Two-Dimensional Molybdenum Ditelluride Well below the Mott Transition**

Zhen Wang^1,2,3†^, Hao Sun^1,2,3†^, Qiyao Zhang^1,2,3^, Jiabin Feng^1,2,3^, Jianxing Zhang^1,2,3^, Yongzhuo Li^1,2,3^, and Cun-Zheng Ning^1,2,3,4*^

^1^Department of Electronic Engineering, Tsinghua University, Beijing 100084, China

^2^Frontier Science Center for Quantum Information, Beijing 100084, China

^3^Beijing National Research Center for Information Science and Technology, Beijing 100084, China

^4^School of Electrical, Computer, and Energy Engineering, Arizona State University, Tempe, AZ 85287, USA

†These authors contributed equally to this work.

*Correspondence to: cning@tsinghua.edu.cn; cning@asu.edu

**This file includes:**

Supplementary text: Sections **S1** to S**10**

Figures **S1** to **S13**

Tables **S1** to **S2**

**Supplementary Text**

**S1. Reflectance measurement and determination of absorption and gain**

**S2. Influence of substrates with metal and modelling of differential reflectance**

**S3. Co-existence and mutual conversion of excitonic complexes**

**S4. Relationship between signal enhancement and optical gain**

**S5. Linear scaling of PL with pumping**

**S6. Formation of trions in 2D materials**

**S7. Simulation of trion gain spectra**

**S8. Determination of pump-induced carrier density, trion density, and gate-controlled electron density**

**S9. Effects of defects and excitonic absorption on the optical gain of trions**

**S10. Further results on optical gain for other gate voltages or devices**

**S1. Reflectance measurement and determination of absorption and gain**

**Figure S1 |** Schematic of the simplified “three-layer” system for reflectance measurement.

Reflectance spectroscopy is widely used to obtain the optical property of a thin film with thickness *d* much smaller than the wavelength *λ*. The relative reflectivity change is mainly due to the existence of the absorptive thin film. Thus, this method can be used to directly characterize the absorption properties of the material. Here, we consider a simplified three-layer material system, as shown in Fig. S1, which consists of a thin MoTe_2_ film (refractive index of$n_{2}$) placed between air ($n_{1}=1$) and an effective substrate ($n_{3}$). For normal incidence with input *I*_0_, the Fresnel coefficients at the air/MoTe_2_ and MoTe_2_/substrate interfaces are defined as

and , respectively, where $\hat{n}_{i}$ is the complex refractive index($\mu_{i}=1$ for non-magnetic materials) and$\hat{\varepsilon}_{i}$is the complex dielectric constant $\hat{\varepsilon}_{i}=\varepsilon_{i}'+i\varepsilon_{i}"$($\varepsilon_{i}'=n_{i}^{2}-k_{i}^{2}$and $\varepsilon_{i}"=2n_{i}k_{i}$). For thin film thickness$d<<\lambda$, the reflection coefficient from the thin film in the first-order approximation is defined as

$\gamma_{123}=\frac{\gamma_{12}+\gamma_{23}e^{-i2\phi}}{1+\gamma_{12}\gamma_{23}e^{-i2\phi}}\approx\frac{\gamma_{12}+\gamma_{23}\left(1-i2\phi\right)}{1+\gamma_{12}\gamma_{23}\left(1-i2\phi\right)}$, where *φ* () is the phase change.

If no thin film exists, then for a two-layer material system, the coefficient of reflection directly from the substrate is defined as $\gamma_{13}=\gamma_{123}\left(d=0\right)=\frac{\gamma_{12}+\gamma_{23}}{1+\gamma_{12}\gamma_{23}}$.

The reflectivity ($R=\left|\left.\gamma\right|\right.^{2}$) ratio is defined by normalizing the signal reflected from the thin film on the substrate to that from the bare substrate without the thin film, which can be expressed in the following form (using the notations in the main text) ^1^:

$\frac{R\left(0,p,s\right)}{R\left(0,p,0\right)}=\frac{R\left(d\right)}{R\left(0\right)}=\left|\left.\frac{\gamma_{123}}{\gamma_{13}}\right|^{2}\right.=1+\frac{8\pi dn_{1}}{\lambda}\frac{\varepsilon_{2}"\left(\varepsilon_{3}'-1\right)-\varepsilon_{3}"\left(\varepsilon_{2}'-1\right)}{\left(\varepsilon_{3}'-1\right)^{2}+\varepsilon_{3}"^{2}}$.

Thus, the differential reflectance can be described as

$\frac{\Delta R}{R}=\frac{R\left(0,p,s\right)-R\left(0,p,0\right)}{R\left(0,p,0\right)}=\frac{R\left(d\right)-R\left(0\right)}{R\left(0\right)}=\frac{8\pi d}{\lambda}\frac{\varepsilon_{2}"\left(\varepsilon_{3}'-1\right)-\varepsilon_{3}"\left(\varepsilon_{2}'-1\right)}{\left(\varepsilon_{3}'-1\right)^{2}+\varepsilon_{3}"^{2}}$. (S1)

For thin film MoTe_2_, we define $\hat{\varepsilon}_{2}=\varepsilon_{b}+\varepsilon_{b}\hat{\chi}$, where $\varepsilon_{b}$ is the background dielectric constant and $\hat{\chi}$is the complex susceptibility. Thus, we have $\varepsilon_{2}'=\varepsilon_{b}+\varepsilon_{b}\chi'$, $\varepsilon_{2}"=\varepsilon_{b}\chi"$and $n_{\text{b}}=\sqrt{\varepsilon{}_{\text{b}}}$. Equation (S1) can then be rewritten as

$\begin{matrix} \frac{\Delta R}{R}=\frac{8\pi d}{\lambda}\frac{\varepsilon_{\text{b}}\chi"\left(\varepsilon_{3}'-1\right)-\varepsilon_{3}"\left(\varepsilon_{\text{b}}+\varepsilon_{\text{b}}\chi'-1\right)}{\left(\varepsilon_{3}'-1\right)^{2}+\varepsilon_{3}"^{2}} \\ =\frac{8\pi d}{\lambda}\frac{\varepsilon_{\text{b}}\chi"\left(\varepsilon_{3}'-1\right)-\varepsilon_{\text{b}}\chi'\varepsilon_{3}"-\varepsilon_{3}"\left(\varepsilon_{\text{b}}-1\right)}{\left(\varepsilon_{3}'-1\right)^{2}+\varepsilon_{3}"^{2}}=\text{A}\chi"+\text{B}\chi'+\text{C} \end{matrix}$, (S2)

where $\text{A}=\frac{8\pi d}{\lambda}\frac{\varepsilon_{\text{b}}\left(\varepsilon_{3}'-1\right)}{\left(\varepsilon_{3}'-1\right)^{2}+\varepsilon_{3}"^{2}}$, $\text{B}=\frac{8\pi d}{\lambda}\frac{-\varepsilon_{\text{b}}\varepsilon_{3}"}{\left(\varepsilon_{3}'-1\right)^{2}+\varepsilon_{3}"^{2}}$, and$\text{C}=\frac{8\pi d}{\lambda}\frac{-\varepsilon_{3}"\left(\varepsilon_{\text{b}}-1\right)}{\left(\varepsilon_{3}'-1\right)^{2}+\varepsilon_{3}"^{2}}$. The A, B, and C coefficients are wavelength-dependent factors mainly determined by the dielectric constant of the effective substrate. If the effective substrate underneath MoTe_2_ is dielectric, *e.g.*, a transparent substrate, then by setting$\varepsilon_{3}"=0$, we have

$\frac{\Delta R}{R}=\text{A}\chi"=\frac{8\pi d}{\lambda}\frac{\varepsilon_{\text{b}}\chi"}{\varepsilon_{3}'-1}=\frac{4n_{\text{b}}d}{n_{3}^{2}-1}\cdot\alpha_{2}$, (S3)

where we have used the definition of the absorption coefficient $\alpha_{2}=\frac{2\pi n_{\text{b}}\chi"}{\lambda}$. Thus, the differential reflectance without pumping is proportional to the intrinsic absorption of the thin film, or

$\left(\frac{\Delta R}{R}\right)_{0}\propto\alpha\left(0\right)$. This is the basis of the measurement of the absorption of a thin film placed on top of a dielectric substrate through differential reflectance spectroscopy.

When we introduce continuous-wave (CW) pumping, $e$, in the measurement, an expression similar to equation (S1) can be obtained. The only difference is that the reflectance $R\left(e,p,s\right)$now contains the PL signal, which has to be subtracted from the reflectance difference, and the differential reflectance is now given by

$\frac{\Delta R}{R}=\frac{R_{e}\left(d\right)-R_{e}\left(0\right)}{R_{e}\left(0\right)}=\frac{\left[R\left(e,p,s\right)-R\left(e,0,s\right)\right]-R\left(e,p,0\right)}{R\left(e,p,0\right)}=\frac{\left[R\left(e,p,s\right)-R\left(e,0,s\right)\right]-R\left(0,p,0\right)}{R\left(0,p,0\right)}$,

where $R\left(e,0,s\right)$ represents the contribution of PL emission to the measured signal, and we have set $R\left(e,p,0\right)\equiv R\left(0,p,0\right)$ since the reflectance signal from the substrate is the same with and without excitation. Thus, we have

$\left(\frac{\Delta R}{R}\right)_{e}\propto\alpha(e)=-g\left(e\right)$. (S4)

Equation (S4) is the basis for determining the optical gain through the measurement of the differential reflectance under CW pumping.

**S2. Influence of substrates with metal and modelling of differential reflectance**

**Influence of metal on the validity of the measurement method**

If the effective substrate is strongly absorbing, such as another semiconductor or a metal, then the relationship between the differential reflectance and optical gain/absorption is slightly different. In our experiment, for the electrically gated devices, there are nominally ~ 50 nm h-BN, ~ 50 nm Au, ~ 30 nm Ti and 300 nm SiO_2_ underneath MoTe_2_, as depicted in Fig. 1 (a) in the main text. To investigate the influences of the metal and determine the validity of equation (S2), we treated the h-BN, Au and Ti tri-layer as an equivalent substrate with an effective refractive index of $n_{3}$, as shown in Fig. S1. Using the Fresnel equation, the reflection coefficient $r_{0}$ from the effective substrate can be calculated using the complex reflective index of h-BN, Au and Ti (see Fig. S2a), with $r_{0}=\frac{1-\hat{n}_{\text{eff}}}{1+\hat{n}_{\text{eff}}}$ and $\hat{n}_{\text{eff}}=\sqrt{\hat{\varepsilon}_{\text{eff}}}$. The determined effective dielectric constant, shown in Fig. S2b, is compared to that of Au. Both the real and imaginary parts of the effective dielectric constant are significantly reduced from those of Au by a factor of approximately 10 in the presence of the ~ 50 nm h-BN layer on top.

**Figure S2 |** **a.** Comparison of the dielectric constants of Au and Ti, and **b**. determined effective dielectric constant of the simplified effective substrate (h-BN/Au/Ti tri-layer).

The complex dielectric function of MoTe_2_ was modelled using a superposition of several Lorentzian oscillators as follows ^2,3^:

$\hat{\varepsilon}\left(\omega\right)=\varepsilon'\left(\omega\right)+i\varepsilon"\left(\omega\right)=\varepsilon_{\text{b}}\left(\infty\right)+\sum_{k=1}^{N} \frac{f_{k}\omega_{k}^{2}}{\omega_{k}^{2}-\omega^{2}-i\omega\Gamma_{k}}$. (S5)

Here, $\varepsilon_{b}\left(\infty\right)$ is an offset dielectric constant at frequencies where there are no excitonic effects, same as the definition in Section S1. $f_{k}$, $\omega_{k}$, and $\Gamma_{k}$ denote the oscillator strength, resonant (peak) energy, and damping factor (full width at half maximum, FWHM) of each optical transition.

$\varepsilon'\left(\omega\right)=\varepsilon_{\text{b}}+\sum_{k=1}^{N} f_{k}\omega_{k}^{2}\frac{\left(\omega_{k}^{2}-\omega^{2}\right)}{\left(\omega_{k}^{2}-\omega^{2}\right)^{2}+\omega^{2}\Gamma_{k}^{2}}$ (S6)

and

$\varepsilon"\left(\omega\right)=\sum_{k=1}^{N} f_{k}\omega_{k}^{2}\frac{\omega\Gamma_{k}}{\left(\omega_{k}^{2}-\omega^{2}\right)^{2}+\omega^{2}\Gamma_{k}^{2}}$ (S7)

are the real and imaginary parts of the dielectric constant, respectively. For bilayer MoTe_2_ in the wavelength range of 1-1.2 eV, three Lorentzian oscillators are typically used to model the dielectric function, including a trion (T) and two excitons (X_1_ and X_2_). The optimized fitting parameters for a representative spectrum are summarized in Table S1 below.

Table S1. Fitting parameters for the complex dielectric function of MoTe_2_.

|  | **Oscillator strength** | **Resonant energy (eV)** | **FWHM (eV)** |
| --- | --- | --- | --- |
| T | 0.023 | 1.132 | 0.01 |
| X_1_ | 0.170 | 1.149 | 0.004 |
| X_2_ | 0.180 | 1.144 | 0.0045 |

$\varepsilon_{\text{b}}$ is determined to be 20 based on the best fit. Now, we have all the parameters needed to calculate A, B, and C, which are plotted in Fig. S3a. As can be seen, within the wavelength range of 0.8-1.8 eV, the B and C values are almost constant and insensitive to the wavelength due to the much smaller value of $\varepsilon_{3}"$ compared to $\varepsilon_{3}'$ (on the order of 25 to 1, as can be seen from Fig. S2b).

We also observe that the effective refractive indices of Au and Ti do not have resonance features in the wavelength range of interest that we measured and vary slowly with frequency, as shown in the inset of Fig. S3a and Fig. S2a. Figure S3b shows the ratio of A and B as a function of photon energy, further verifying that the B coefficient is much smaller than A. Figure S3c plots the values of $\text{A}\chi"$,$\text{B}\chi'$and $\text{C}$. As can be seen, $\text{B}\chi'$does not influence the spectral features very much, and$\text{C}$only adds a relatively flat background to the reflectance spectrum without any resonance features. The reflectance spectrum calculated using only the $\text{A}\chi"$ term can reproduce the features calculated using $\text{A}\chi"+\text{B}\chi'+\text{C}$quite well, as shown in Fig. S3d. Thus, the reflectance contrast can be simplified as

$\frac{\Delta R}{R}=\text{A}\chi"+\text{B}\chi'+\text{C}\approx\text{A}\chi"\propto\alpha_{2}$, (S8)

Alternatively, we can consider that the differential reflectance determines the absorption coefficient or gain up to a constant background as follows:

$\frac{\Delta R}{R}-\text{C}\approx\text{A}\chi"\propto\alpha_{2}$. (S9)

**Figure S3 | a**. A, B, and C coefficients calculated based on the simplified effective substrate (h-BN/Au/Ti tri-layer). **b**. Calculated A/B ratio. The inset shows a zoomed-in view in the energy range of 1-1.25 eV. **c**. Reflectance spectra calculated using $\text{A}\chi"$,$\text{B}\chi'$and $\text{C}$. **d**. Reflectance spectra calculated using $\text{A}\chi"$ and $\text{A}\chi"+\text{B}\chi'+\text{C}$.

One of the important requirements in the measurement of differential reflectance is the measurement of both $R\left(0,p,s\right)$ and $R\left(0,p,0\right)$ under exactly the same conditions as much possible with the only difference being whether the sample is present or not. However, in reality, the measurement of these two quantities have to be performed at two different locations of the substrates (see Fig. S1) and at two different time windows. Due to the unavoidable differences between the topography and other physical conditions of the two locations of the substrate and temporal fluctuations of the probe light, it is very challenging to guarantee that the only difference between the measurements of the two quantities is the presence or absence of the sample. Another factor is the height difference between the two locations since an ~ 10 nm thick h-BN layer is also present for the sample (see the details of the sample structure). This could lead to differences in the focal planes at the two locations. Other factors include changes in the roughness or air gap across the substrate. As an example, Fig. S4a compares $R\left(0,p,0\right)$ and $R\left(0,p,s\right)$, where we can clearly see that there is a shift of the two quantities (black and blue curves). The difference is clearly non-physical since at the low-frequency end of the spectrum well below any physical absorption features, the trends of the two spectra are expected to be identical. The discrepancy is a result of a combination of the possible factors described above. To account for such an artificial difference, we scaled $R\left(0,p,0\right)$ by a factor, A, such that $\text{A}*R\left(0,p,0\right)$ agrees with $R\left(0,p,s\right)$ at the low-frequency end of the spectrum, where no absorption of the sample is expected. In the specific example shown in Fig. 4a, $R\left(0,p,0\right)$ is scaled by a factor of A = 0.844, shown as the dotted red curve. The fact that the two curves agree almost perfectly at the low-frequency end of the spectrum again indicates that the initial difference is not physical. The difference at higher photon energies is the result of absorption changes due to the presence of trions and excitons. The differential reflectance spectra processed with and without scaling $R\left(0,p,0\right)$ are compared in Fig. S4b, where we can see that the differential reflectance without scaling of $R\left(0,p,0\right)$ (blue curve) shows an unphysical negative background. Otherwise, the two spectra agree with each other in the real features of physical relevance. It is important to emphasize that for the processing of differential reflectance spectra under various pumping levels, we only need to perform such scaling once for $R\left(0,p,0\right)$ with respect to $R\left(0,p,s\right)$, not $R\left(e,p,s\right)$. All the absorption or differential reflectance spectra shown in the main text are processed in this way to avoid the unphysical negative background.

**Figure S4 | a**. Reflected intensities of $R\left(0,p,0\right)$, $R\left(0,p,s\right)$ and $\text{A}*R\left(0,p,0\right)$. **b**. Calculated differential reflectance with and without scaling of $R\left(0,p,0\right)$.

**Modelling of differential reflectance spectra**

To further investigate the effect of the complex structure (considering the SiO_2_/Si substrate, as shown in Fig. 1(a) in the main text) on the results of the reflectance measurement, we used the standard transfer matrix method (TMM) to theoretically analyse the data. For a given stack of thin multilayer media with known complex refractive indices and thicknesses, TMM was used to calculate the reflectance $R$ and transmittance $T$of electromagnetic waves through the multilayer structure. The absorbance of each layer could then be obtained from$A=1-T-R$. The simulation was carried out using an open-source MATLAB code ^4^. For simplicity, we assumed that each layer plane perpendicular to the incidence direction is infinite and flat, the material is homogeneous and isotropic, and the injected plane wave is a continuous wave. For the complex multilayer structure shown in Fig. 1 (a) in the main text, the top ambient part and the bottom Si substrate were considered semi-infinite. For the MoTe_2_ film, equations (S5-S7) were used together with the optimized parameters listed in Table S1. The thicknesses of MoTe_2_ were 0.7 nm and 1.4 nm for mono- and bilayer samples, respectively. The other complex indices of refraction of h-BN ^5^, Au ^6^, Ti ^7^, SiO_2_ ^8^ and Si ^9^ were obtained from the literature.

We modelled the differential reflectance spectra by comparing several situations: the simplified “equivalent substrate” (h-BN/Au/Ti tri-layer) with and without the top h-BN layer and the exact complex layer stack shown in Fig. 1 (a) in the main text. The modelled differential reflectance spectra are shown in Fig. S5 for the three cases. As can be seen, the three cases only differ by a constant background with no effect on the main resonance features. These results further affirmed that the details of the substrate layer structures do not affect the resonant features of the absorption and gain of MoTe_2_. The measured differential reflectance spectrum can reproduce the absorption/gain property of MoTe_2_, largely independent of the details of the substrate.

**Figure S5 | a**. Simulated differential reflectance spectra for bilayer MoTe_2_ in three situations: simplified equivalent substrate as discussed in Fig. S2b (black); adding a top h-BN layer (of ~ 10 nm thickness) to the simplified structure (red); and the full layer structure shown in Fig. 1 (a) in the main text (blue).

**S3. Co-existence and mutual conversion of excitonic complexes**

To clearly display the co-existence and mutual conversion of various excitonic complexes as the gate voltage evolves, stacked line plots of the same data set shown in Fig. 1 c, d, e, f in the main text are shown in Fig. S6 a, b, c, d, respectively. For the bilayer sample, as shown in Fig. S6a, the charge neutral voltage (CNV) is at ~ -2.5 V, where the trion emission shows a minimum. The negative sign of the CNV suggests that the sample was initially electron-doped. This is true for all the samples we measured. Excitons (X_1_ and X_2_) can co-exist and mutually convert with a change in the relative intensity as the voltage is increased relative to the CNV. The PL intensities for the electron-trion (T^-^) and hole-trion (T^+^) states are dominant at higher gate voltages, which induce more electrostatic carriers to form trions. The absorption features of both excitons can be observed throughout the whole voltage range, while the features of trions become prominent only at high gate voltages, as shown in Fig. S6b. The binding energies of T^-^ and T^+^ are ∼9 meV and ~ 14 meV with respect to X_2_ and X_1_, respectively. For the monolayer sample, as shown in Fig. S6c and Fig. S6d, the CNV is at ~ -1 V, where both the exciton emission and absorption show maxima. The exciton emission decreases with increasing voltage in both directions due to the formation of more trions with more available electrons or holes. At high voltages, no exciton emission can be clearly seen in the PL spectra, and only trion emission dominates, indicating an almost full conversion of excitons into trions. T^-^ and T^+^ exhibit symmetric features with respect to the CNV. The binding energies of T^-^ and T^+^ are identical at ∼22 meV, indicating nearly equal effective masses for electrons and holes in 2D MoTe_2_.

**Figure S6 |** Gated PL (**a**) and absorption (**b**) spectra for the bilayer sample. **c. & d.** Same measurements for the monolayer sample. The optical pumping for (a) and (c) is 5 µW and 10 µW, respectively.

**S4. Relationship between signal enhancement and optical gain**

Signal enhancement (SE) is sometimes referred to as on-off gain. It describes the ratio of the output signals with and without pump excitation. Thus, it is defined as the absorption change with and without excitation. Using the definitions in Section S1 and the same notations as in the main text, SE can be expressed as

$\begin{matrix} \text{SE}=\left(\frac{\Delta R}{R}\right)_{e}-\left(\frac{\Delta R}{R}\right)_{0}=\frac{R\left(e,p,s\right)-R\left(e,0,s\right)-R\left(0,p,0\right)}{R\left(0,p,0\right)}-\frac{R\left(0,p,s\right)-R\left(0,p,0\right)}{R\left(0,p,0\right)} \\ \frac{R\left(e,p,s\right)-R\left(e,0,s\right)-R\left(0,p,\text{s}\right)}{R\left(0,p,0\right)}\propto g\left(e\right)+\alpha\left(0\right) \end{matrix}$. (S10)

The above equation is the well-known relationship between the signal enhancement (or pump-induced reduction or bleaching of absorption) and optical gain. It is clear that one has to subtract the material absorption from the signal enhancement to obtain the optical gain, and the signal enhancement is typically larger than the material gain. Optical gain can occur only when the signal enhancement is larger than the intrinsic absorption of the material. Moreover, the signal enhancement is equal to the optical gain when the absorption at zero pumping is zero at a given wavelength. In our experiments, the gain and absorption features of the trion are well separated in the frequency domain. We can obtain the gain spectrum from the SE spectrum by subtracting the background at frequencies where there is no absorption (or where no absorption is believed to occur based on physical arguments). The absorption and signal enhancement results for the bi- and monolayer samples are shown in Fig. S7, where the SE is obtained from equation (S10). At low excitation levels, SE is close to zero. With increasing pump power, SE shows a monotonous increment. By further increasing pumping, SE starts to show saturation due to the heating effect induced by CW laser pumping and saturation of the optical gain as a result of the finite electron doping density. We note that there is some slight bleaching of the exciton absorptions in the case of the bilayer, but no bleaching is observed in the case of the monolayer. The SE shows maxima near the trion gain peak, consistent with the occurrence of optical gain, as shown in Fig. 2 in the main text.

**Figure S7 |** Absorption and signal enhancement results for **(a)** bilayer and **(b)** monolayer MoTe_2_ samples, corresponding to Fig. 2 in the main text.

**S5. Linear scaling of PL with pumping**

To help verify the origin of the optical gain, we plot the integrated PL intensities of different excitonic complexes as a function of pump excitation in Fig. S8, including electron-trion, hole-trion, and two exciton complexes, for the same samples as presented in Fig. 2 in the main text. The respective scaling index (S) of the PL intensity with pumping for each excitonic complex at different voltages is labelled. For the monolayer sample, exciton emission is only observable at a gate voltage of -1 V, near the CNV. The PL spectrum is dominated by trion emission at higher gate voltages. All the plotted curves show a nearly linear dependence, with S close to one, excluding the possibility of PL emission from exciton-exciton scattering or biexcitons, which show a quadratic PL dependence on pumping. Within the range of pumping, no biexciton emission is observed. Only excitons and trions show a linear dependence of the PL intensity on pumping.

**Figure S8 |** Log-log plot **(a)** and linear plot **(b)** of integrated PL intensity measured at 4 K and gate voltages of +/- 10 V versus pump excitation for the same bilayer sample as presented in Fig. 2 (a,b,c) in the main text. **c. & d.** Similar plots for the same monolayer sample as in Fig. 2 (d,e,f) in the main text.

**S6. Formation of trions in 2D materials**

Figure S9a shows the calculated band structure of MoTe_2_ for the K valley. In 2D TMDCs, there are typically three types of negative trions and one type of positive trion associated with each of the K and K’ valleys due to the much larger spin splitting of valence bands, as explained in detail in the literature ^10,11^. Figure S9 represents three types of negative trions, including two types of inter-valley trions (Fig. S9b and Fig. S9c) and one intra-valley trion (Fig. S9d). In conventional III-V or II-VI semiconductors, trions consist of three particles that are more equal due to the similar binding energies of trions and excitons, whereas in 2D material systems, the larger difference in the binding energies of excitons and trions (~ 600 meV and ~ 20 meV, respectively, in MoTe_2_) makes a trion more similar to a bound state formed between an exciton and a charged particle (an electron or a hole). Since the creation of an exciton does not involve any change in momentum, the momentum of a trion is the same as that of the participating charged particle. The only difference is that the effective mass is now equal to $m_{\text{t}}=2m_{\text{e}}+m_{\text{h}}$ for a negative trion or $m_{\text{t}}=m_{\text{e}}+2m_{\text{h}}$ for a positive trion. Thus, a simplified “two-band” model for optical transitions involving a negative trion is schematically represented in Fig. 3 (a) in the main text. The absorption or emission of a photon occurs between an electronic state and a trionic state and will cause no change in momentum.

**Figure S9 |** **a**. Schematic of the electronic band structure of MoTe_2_ for the K valley. ∆c and ∆v are the splittings of the conduction band and valance band, respectively. The arrows indicate the spin directions. Schematic of negative trion compositions, including inter-valley trions (**b & c**) and an intra-valley trion (**d**). Energies are not to scale for clarity.

**S7. Simulation of trion gain spectra**

Using Fermi's golden rule, for semiconductors reaching a quasi-equilibrium state under optical pumping, the optical gain spectrum can be expressed as follows:

$G(\omega)=C_{0}\int_{0}^{\infty} \left|\left.\mu\left(k\right)\right|\right.^{2}\cdot\rho_{\text{r}}\left(f_{\text{c}}-f_{\text{v}}\right)\cdot L\left(k,\omega\right)dk$, (S11)

where $C_{0}$ is the gain co-factor, $\left|\left.\mu\left(k\right)\right|\right.^{2}$is the optical dipole matrix element, $\rho_{\text{r}}$is the density of states, and $f_{\text{c}}$ and$f_{\text{v}}$ are the Fermi distributions of electrons and holes, respectively. $L\left(k,\omega\right)$ is a lineshape function, where $k$is the momentum and $\omega$ is the resonant frequency. This expression can be extended to describe the absorption/gain properties in 2D material systems, which can be considered as a simplified “two-band” model. It should be noted that the 2D joint density () is independent of momentum. Thus, the trion gain spectrum can be rewritten as (same as equation (3) in the main text)

$G(\omega)\propto\sum_{k} \left|\left.\mu\left(k\right)\right|\right.^{2}\cdot L\left(k,\omega\right)\cdot\left[f_{\text{t}}\left(k\right)-f_{\text{e}}\left(k\right)\right]$. (S12)

The situation is very similar to the inter-subband absorption process in semiconductors, in which the electron energies in the initial conduction band and the final trion band are and (measured from the bottom of the conduction band), respectively, where $E_{\text{t}}$is the trion peak energy, and $m_{\text{t}}=2m_{\text{e}}+m_{\text{h}}$ for electron trions. For a given density of electrons ($n_{\text{e}}$) and trions ($n_{\text{t}}$), their chemical potentials are determined by

and, respectively. The Fermi-Dirac distribution is then expressed as $f(k)=\frac{1}{1+e^{\left(E-\mu\right)/k_{B}T}}$.

We assume a Gaussian lineshape with a homogeneous broadening linewidth of $FWHM=2\gamma$, or .

Figure S10a shows the chemical potentials for both electrons and trions as a function of the pump-induced carrier density. Figure S10b plots the electron and trion occupation probability functions for both the Fermi-Dirac and Maxwell-Boltzmann distribution functions compared at T = 10 K for a carrier density of ~ 7.2 × 10^7^ cm^-2^. This density represents the typical levels of carriers in our experiment. The results for the two distribution functions are identical for both electrons and trions over the range of positive energies where carriers are populated with a non-zero density of states. Thus, both of the carrier distributions can be approximated by the Boltzmann statistics. The larger effective mass for trions also causes a separation between $E_{\text{T}}$ and $E_{\text{e}}$ orders of magnitude larger than $k_{B}T$, and the absorption gain crossover can be obtained as ; thus, degeneracy is actually not critical to achieve trion gain ^12^. It should be noted that we did not consider the k dependence of the optical dipole matrix element in the modelling process of the trion gain spectra.

**Figure S10 |** **a.** Chemical potential for both electrons and trions versus pump density. **b.** Occupation probability of both electrons and trions at T = 10 K for a carrier density of ~ 7.2 × 10^7^ cm^-2^. The horizontal axis “0” refers to the conduction band minimum. The shaded region indicates the positive energy region, where carriers are populated with the 2D density of states and the results for the two distribution functions are almost identical.

**S8. Determination of pump-induced carrier density, trion density, and gate-controlled electron density**

**Estimation of pump-induced carrier density**

The carrier density change due to optical pumping and the finite carrier depletion lifetime are determined by the following rate equation:

. (S13)

The steady-state pump-induced carrier density is thus given by

, (S14)

where $P$is the pump power, $\omega$ is the corresponding frequency, and $L$is the effective absorption length. We define the total absorption as $\alpha L=\alpha\cdot\frac{4n_{\text{b}}d}{\left(n_{3}+1\right)^{2}}$ (%) ^13^, where $d$ is the thickness of MoTe_2_ and $n_{\text{b}}$ and $n_{3}$ are defined in Section S1. $\alpha$and $\tau$are two unknown parameters that represent the total absorption and effective carrier lifetime, respectively. The absorption coefficient at the pump laser wavelength was determined through both reflectance and photoluminescence excitation (PLE) measurements. The detailed process is described as follows: First, we measured the reflectance spectra of MoTe_2_ placed on top of a quartz substrate. By fitting the measured reflectance spectrum using TMM, as discussed in Section S2, we could calculate the absorption coefficient at the peak positions of A and B excitons using equation (S3), as shown in Fig. S11a. Due to the wavelength limit of the light source we used for the reflectance measurement, a separate PLE measurement was conducted for the photon energy range of 1.25 to 2.05 eV to include the wavelength (1.96 eV) of the pump laser in our gain measurement. The PLE spectrum starts at 1.25 eV due to the filter cut-off wavelength being set at the A exciton peak in the measurement setup. By requiring the absorption of the B exciton to be the same in the two independent measurements, the absolute value of absorption can be determined for the entire PLE measurement, as shown on the right vertical axis of Fig. S11b. Finally, we could extract the total absorption at the pump laser wavelength from the PLE curve, obtaining a value of 7 %. $\tau$is typically of several picoseconds for 2D materials ^14^. Now, we have both$\alpha$and$\tau$, and using equation (S14), we can correlate the pump excitation P in the experiment with the photoexcited carrier density$n_{\text{P}}$. For the 633 nm HeNe laser at a pump power of 5 µW, the calculated $n_{\text{P}}$is 3.6$\times$10^7^ cm^-2^.

**Figure S11 |** **a.** Reflection spectrum of MoTe_2_ measured on top of a quartz substrate, showing A and B excitons. **b.** PLE measurement results; the absorption at the pump laser energy was determined to be 7 %.

**Determination of trion density and gate-controlled electron density**

As stated in the trion gain model in the main text (see Fig. 3 (b)), we assume that optical pumping only induces excitons directly or trions. We ignore free/unbound carriers. Then, the total electron and hole densities are $n=n_{\text{e}}+n_{\text{x}}+2n_{\text{t}}$and$p=n_{\text{h}}+n_{\text{x}}+n_{\text{t}}$, respectively. Here, we only consider electron trions for simplicity, which are formed by either two electrons and a hole or one electron and an exciton. The total electrons are generated by optical pumping and doping, whereas the total holes are induced by pumping; thus, we have the following relations: $n=n_{\text{P}}+n_{\text{D}}$ and$p=n_{\text{P}}$, where $n_{\text{D}}$is the electron doping density controlled by the gate voltage and $n_{\text{P}}$is the carrier density generated by optical pumping. Before pumping, the background doping density (induced by defects, gating or doping) is equal to the density of electrons. By applying optical pumping, part of the doped electrons can be incorporated with photoexcited excitons to form trions. This limits the maximum trion density to the doped electron density of$n_{\text{D}}$. If we assume that the free hole density is negligible in the system, $n_{\text{h}}\approx0$, then from charge conservation, the following relations exist: $n_{\text{D}}=n_{\text{e}}+n_{\text{t}}$ and $n_{\text{p}}=n_{\text{x}}+n_{\text{t}}$. The ratio of trions and excitons can be obtained from the mass-action law: $\frac{n_{\text{t}}}{n_{\text{x}}}=\frac{n_{\text{e}}}{K\left(T\right)}$, where is a temperature-dependent equilibrium constant; $m_{\text{X}}=m_{\text{e}}+m_{\text{h}}$ and $m_{\text{T}}=2m_{\text{e}}+m_{\text{h}}$ are the effective masses of an exciton and an electron trion, respectively; and $E_{\text{T}}^{\text{b}}$ is the trion binding energy. Thus, the trion density can be calculated by

$n_{\text{t}}=\frac{n_{\mathrm{P}}+n_{\text{D}}+K\left(T\right)-\sqrt{\left(n_{\mathrm{P}}+n_{\text{D}}+K\left(T\right)\right)^{2}-4n_{\mathrm{P}}n_{\text{D}}}}{2}$. (S15)

The electron doping density $n_{\text{D}}$is then determined by obtaining the best fit to the measured gain spectra using equation (S12) and the calculated trion density. The modelled gain spectra agree well with the measured spectra with a single $n_{\text{D}}$of 7.2$\times$10^7^ cm^-2^. We also solved the rate equation for trions and excitons and carefully extracted$n_{\text{D}}$from the trion spectral weight of the experimental data. Both methods to determine $n_{\text{D}}$agree quite well with each other. The parameters used in the theoretical modelling are summarized in Table S2.

Table S2. Definitions and values of parameters used in theoretical modelling.

| **Parameter** | **Definition** | **Value** |
| --- | --- | --- |
| *S* | Effective area | 12.6 μm^2^ |
| *τ* | Effective carrier lifetime | 4 ps ^14^ |
| *m*_e_ | Electron mass | 0.69m_0_ ^3^ |
| *m*_h_ | Hole mass | 0.66m_0_ ^3^ |
| *T* | Temperature | 4 K |
| *E*_T_^b^ | Trion binding energy | 9 meV |

**S9. Effects of defects and excitonic absorption on the optical gain of trions**

To achieve positive gain, the material quality is important. However, this is very challenging to achieve in practice, especially for exfoliated materials, which degrade during the multiple transfer processes when fabricating electrically gated devices. To discuss in more detail how the material quality could affect the optical gain, here, we compare two samples discussed in the main text, presented in Fig. 2 (a-c) and Fig. 4. The key difference between Fig. 2 (a-c) (sample #70) and Fig. 4 (sample #10) is that Fig. 2 (b-c) shows a flat background with an overall positive trion gain near the trion peak, while Fig. 4 (a-c) shows a gain peak near the trion feature, but with a tilted negative background. For most of the parameter values studied, the trion gain peak is still below zero.

To understand this difference, we theoretically modelled the differential reflectance spectra as discussed in SI Section S2. We chose the same fitted trion linewidth from experiments while choosing the exciton linewidth corresponding to that of sample #70 (FWHM=0.012 eV) or #10 (FWHM=0.03 eV). Fig. S12a shows the comparison, where we see that the increase in the exciton linewidth for sample #10 (with everything else the same) leads to a decrease in the trion gain peak below zero. Due to the close proximity of the exciton and trion peak energies, the increased exciton linewidth increases the spectral overlap between the absorbing excitons and trions with gain. Such coupling in the spectral domain leads to the disappearance of the optical gain of trions. The wider exciton linewidth in sample #10 compared to sample #70 is likely due to increased defects and the associated inhomogeneous broadening of excitons. An increase in defects in #10 compared to sample #70 is consistent with the PL spectral difference between the two samples, as shown in Fig. S12b. Due to the increased defect absorption below and around the trion emission peak, the spectral coupling of defect absorption to trion gain leads to an additional decrease in the trion gain, similar to the coupling to excitons at higher frequency. In general, we can see that the coupling to defect absorption on the lower frequency side and to exciton absorption on the higher frequency side leads to a decrease in the trion gain and the tilted negative background. This leads to the overall negative gain despite the existence of trion gain near the trion feature. This coupling to absorptive features on both frequency sides of the trion gain is illustrated in Fig. S12c.

**Figure S12 | a.** Modelled differential reflectance spectra based on the data at the highest pumping level for the samples in Fig. 2 (a-c) at 10 V and Fig. 4 (a) at 8 V. The exciton linewidth was chosen to correspond to that of sample #70 or sample #10. **b.** Comparison of the PL spectra for the two samples. **c.** Gain spectrum from Fig. 4 (b-c) for sample #10 at 7 V to illustrate the roles of absorptive features on both sides of the trion gain peak.

**S10. Further results on optical gain for other gate voltages or devices**

Figure S13 shows the gain spectra of MoTe_2_ for other gate voltages or devices. The absolute optical gain achieved in the material is mainly determined by the material quality and the electron (hole) doping density produced by gating. We note that throughout this research, a smooth and well-behaved gain peak for samples is always accompanied by less defect-related PL emission. The gain peak becomes noisy and poorly shaped whenever the PL spectrum shows strong defect emission or defect emission over a wide spectral range below the trion peak energy. Even if the material has some imperfections, optical gain can sometimes occur at relatively high gate voltages or pumping levels.

To test the instability of optical gain due to possible material degradation over time, we measured the optical gain of the same sample before and after storage in ambient conditions for three weeks. The results are shown in Fig. S13c and Fig. S13d. As shown, within the time frame that we measured, no significant degradation of the material quality is observed. Optical gain can still be achieved at a slightly higher gate voltage of -12 V. This result indicates that the fabricated MoTe_2_ device shows decent stability of the material quality, which may have benefited from the encapsulation with h-BN for protection.

**Figure S13 |** Gain spectra evolution with increasing pumping level for **(a)** the same bilayer device as in Fig. 2 (a,b,c) in the main text at a gate voltage of -10 V, **(b)** another bilayer device with a similar device structure at 10 V, **(c)** the same monolayer device as in Fig. 2 (d,e,f) in the main text at a gate voltage of 7 V, and **(d)** the same monolayer device as in **(c)** after storage in ambient conditions for three weeks.

**References**

1. McIntyre, J. D. E. & Aspnes, D. E. Differential reflection spectroscopy of very thin surface films. *Surface Science* **24**, 417–434 (1971).

2. Li, Y. *et al.* Measurement of the optical dielectric function of monolayer transition-metal dichalcogenides: MoS_2_ , MoSe_2_ , WS_2_ , and WSe_2_. *Physical Review B* **90**, 205422 (2014).

3. Ramasubramaniam, A. Large excitonic effects in monolayers of molybdenum and tungsten dichalcogenides. *Physical Review B* **86**, 115409 (2012).

4. Burkhard, G. F., Hoke, E. T. & McGehee, M. D. Accounting for interference, scattering, and electrode absorption to make accurate internal quantum efficiency measurements in organic and other thin solar cells. *Advanced Materials* **22**, 3293–3297 (2010).

5. Lee, S.-Y., Jeong, T.-Y., Jung, S. & Yee, K.-J. Refractive index dispersion of hexagonal boron nitride in the visible and near‐infrared. *Physica Status Solidi B* **256**, 1800417 (2019).

6. Yakubovsky, D. I., Arsenin, A. V., Stebunov, Y. V., Fedyanin, D. Y. & Volkov, V. S. Optical constants and structural properties of thin gold films. *Optics Express* **25**, 25574–25587 (2017).

7. Johnson, P. & Christy, R. Optical constants of transition metals: Ti, V, Cr, Mn, Fe, Co, Ni, and Pd. *Physical Review B* **9**, 5056–5070 (1974).

8. Gao, L., Lemarchand, F. & Lequime, M. Exploitation of multiple incidences spectrometric measurements for thin film reverse engineering. *Optics Express* **20**, 15734–15751 (2012).

9. Schinke, C. *et al.* Uncertainty analysis for the coefficient of band-to-band absorption of crystalline silicon. [*AIP Advances*](https://aip.scitation.org/journal/adv) **5**, 067168 (2015).

10. Yu, H., Cui, X., Xu, X. & Yao, W. Valley excitons in two-dimensional semiconductors. *National Science Review* **2**, 57–70 (2015).

11. Drüppel, M., Deilmann, T., Krüger, P. & Rohlfing, M. Diversity of trion states and substrate effects in the optical properties of an MoS_2_ monolayer. *Nature Communications* **8**, 2117 (2017).

12. Puls, J. *et al.* Laser action of trions in a semiconductor quantum well. *Physical Review Letters* **89**, 287402 (2002).

13. Li, Y. & Heinz, T. F. Two-dimensional models for the optical response of thin films. [*2D Materials*](https://iopscience.iop.org/journal/2053-1583) **5**, 025021 (2018).

14. Robert, C. *et al.* Excitonic properties of semiconducting monolayer and bilayer MoTe_2_. *Physical Review B* **94**, 155425 (2016).
