# Supplementary material for: Excitonic complexes and optical gain in two-dimensional molybdenum ditelluride well below the Mott transition
Source: Light Sci Appl. 2020 Mar 10;9:39. doi: 10.1038/s41377-020-0278-z (PMC7064520; doi:10.1038/s41377-020-0278-z)
Supplement: Supplementary file 2 — SI figure [file 41377_2020_278_MOESM2_ESM.pdf]

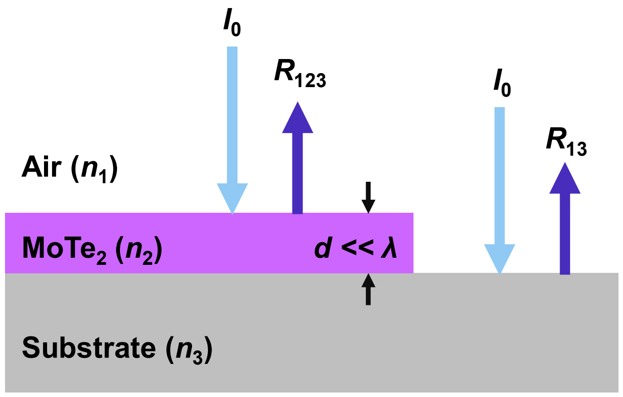

Figure S1 | Schematic of the simplified "three-layer" system for reflectance measurement.

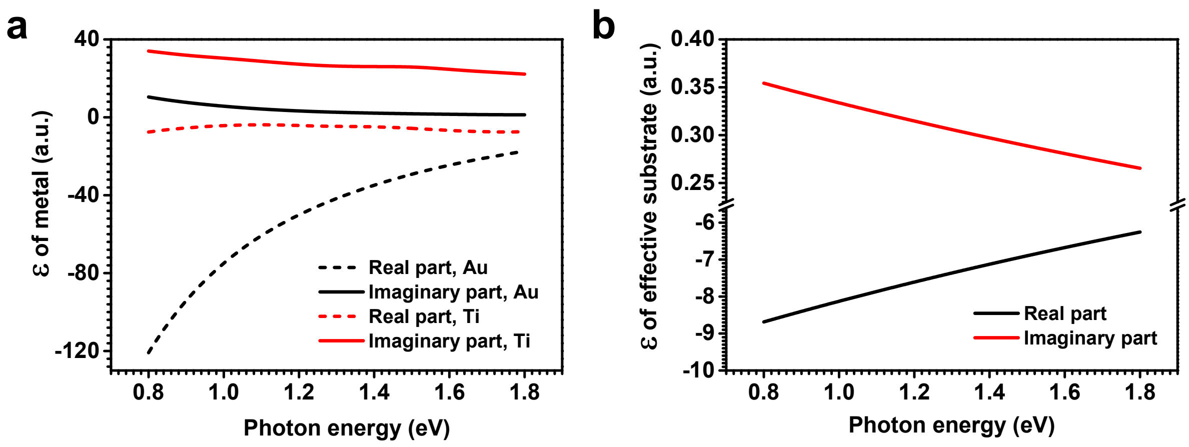

Figure S2 | a. Comparison of the dielectric constants of Au and Ti, and **b**. determined effective dielectric constant of the simplified effective substrate (h-BN/Au/Ti tri-layer).

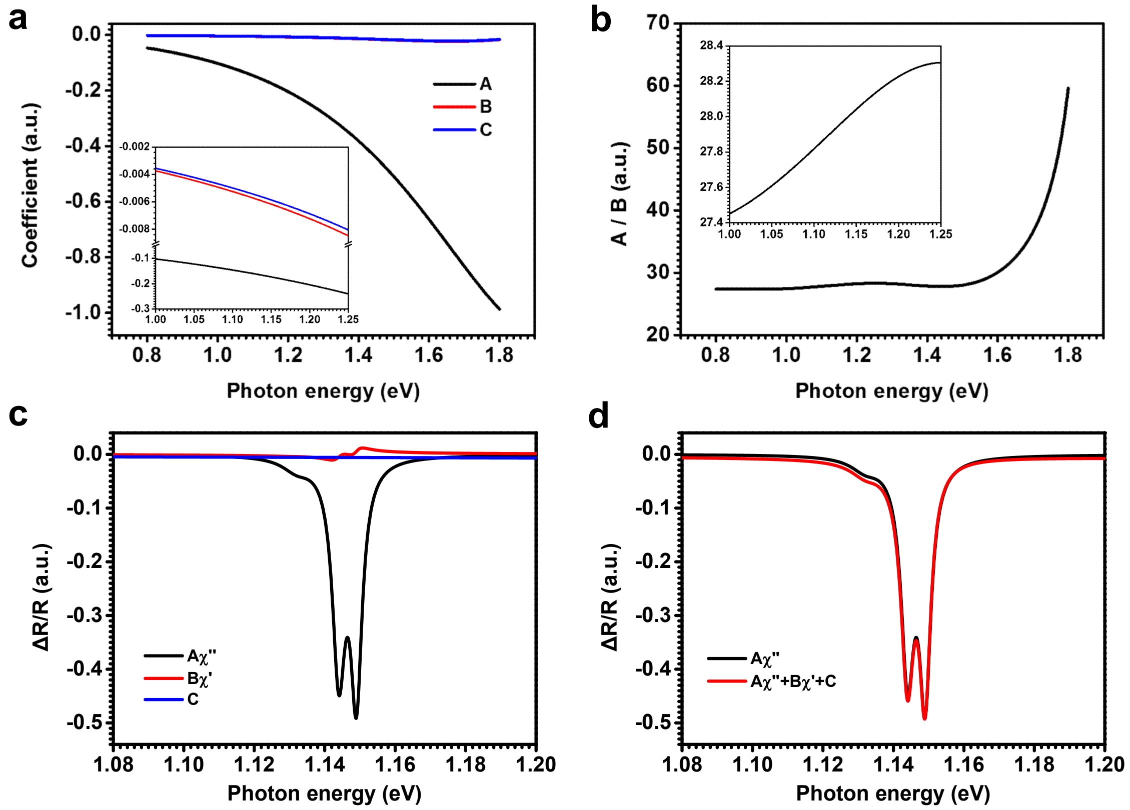

Figure S3 | a. A, B, and C coefficients calculated based on the simplified effective substrate (h-BN/Au/Ti tri-layer). **b**. Calculated A/B ratio. The inset shows a zoomed-in view in the energy range of 1-1.25 eV. **c**. Reflectance spectra calculated using $A\chi''$, $B\chi'$ and C. **d**. Reflectance spectra calculated using $A\chi''$ and $A\chi''+B\chi'+C$.

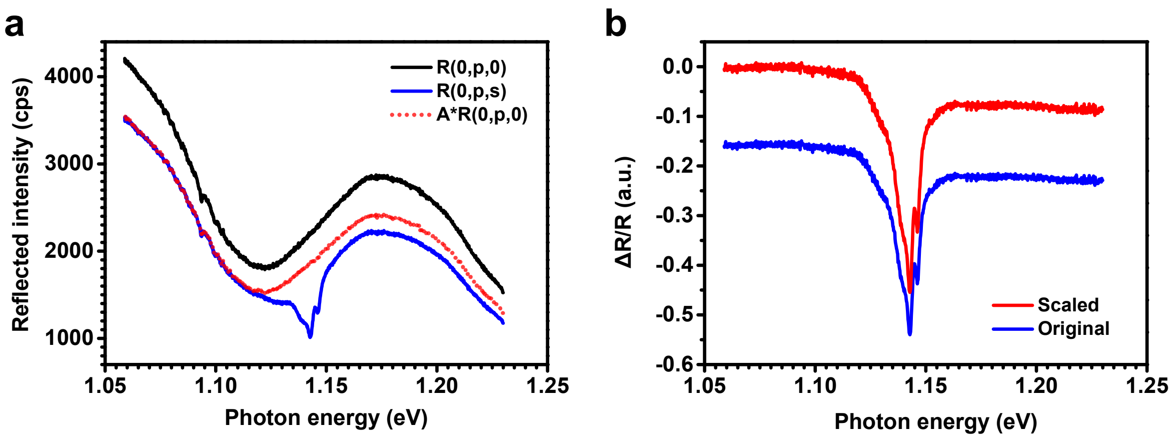

Figure S4 | a. Reflected intensities of $R(0,p,0)$, $R(0,p,s)$ and $A*R(0,p,0)$. **b**. Calculated differential reflectance with and without scaling of $R(0,p,0)$.

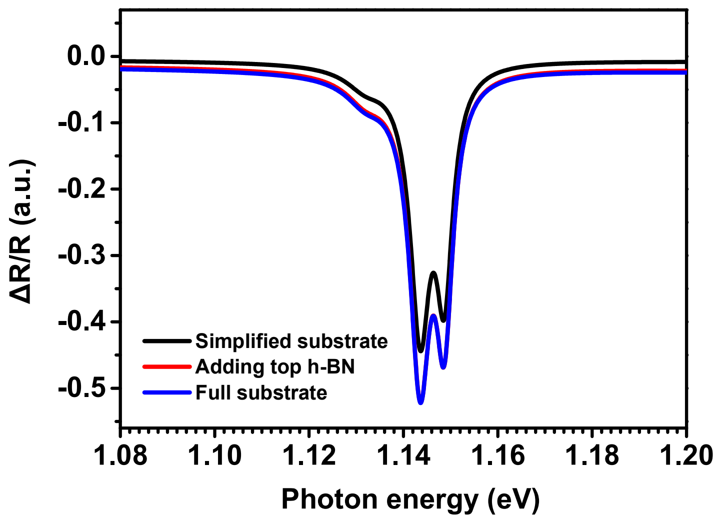

Figure S5 | a. Simulated differential reflectance spectra for bilayer $MoTe_2$ in three situations: simplified equivalent substrate as discussed in Fig. S2b (black); adding a top h-BN layer (of ~ 10 nm thickness) to the simplified structure (red); and the full layer structure shown in Fig. 1 (a) in the main text (blue).

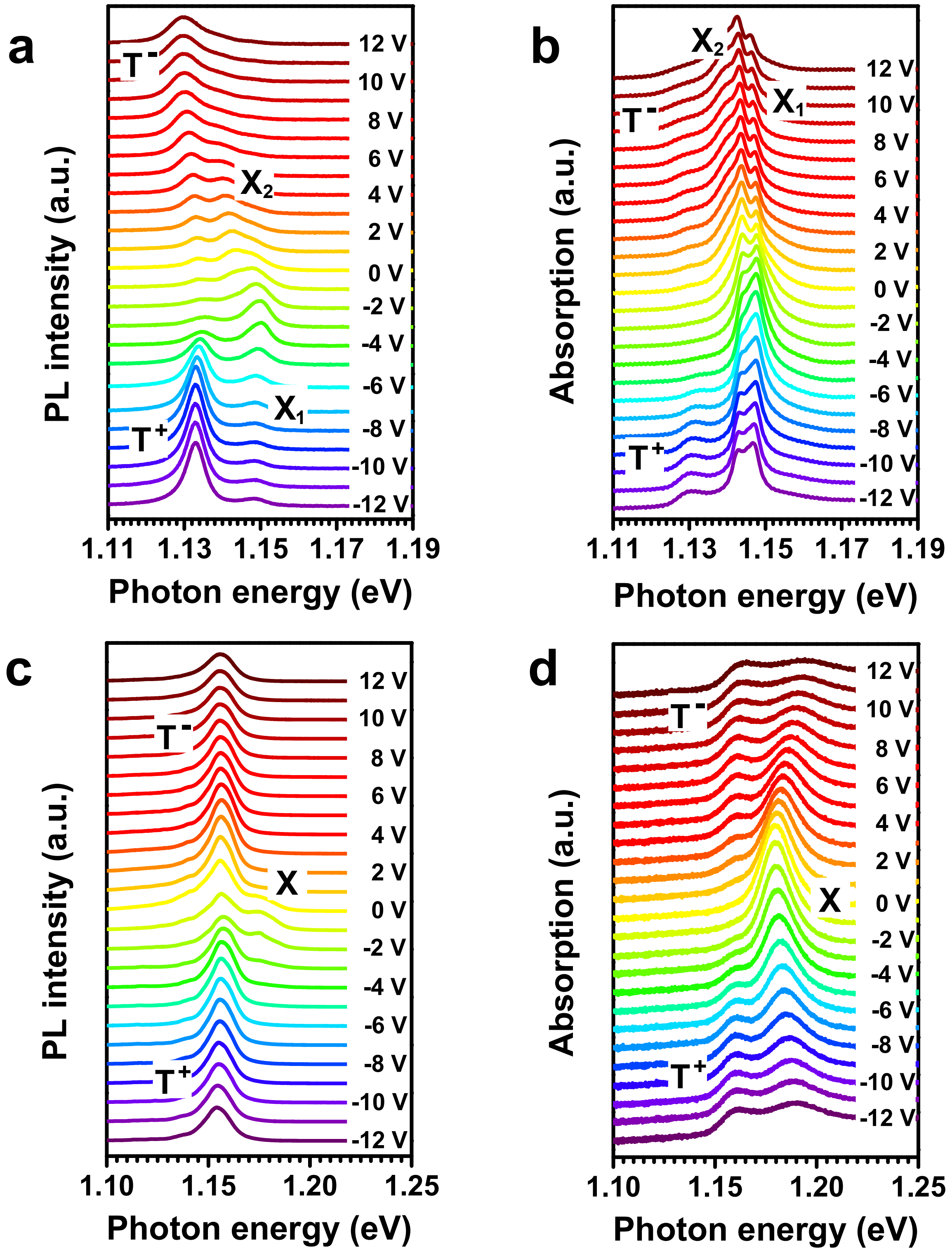

Figure S6 | Gated PL (**a**) and absorption (**b**) spectra for the bilayer sample. **c. & d.** Same measurements for the monolayer sample. The optical pumping for (a) and (c) is 5 μW and 10 μW, respectively.

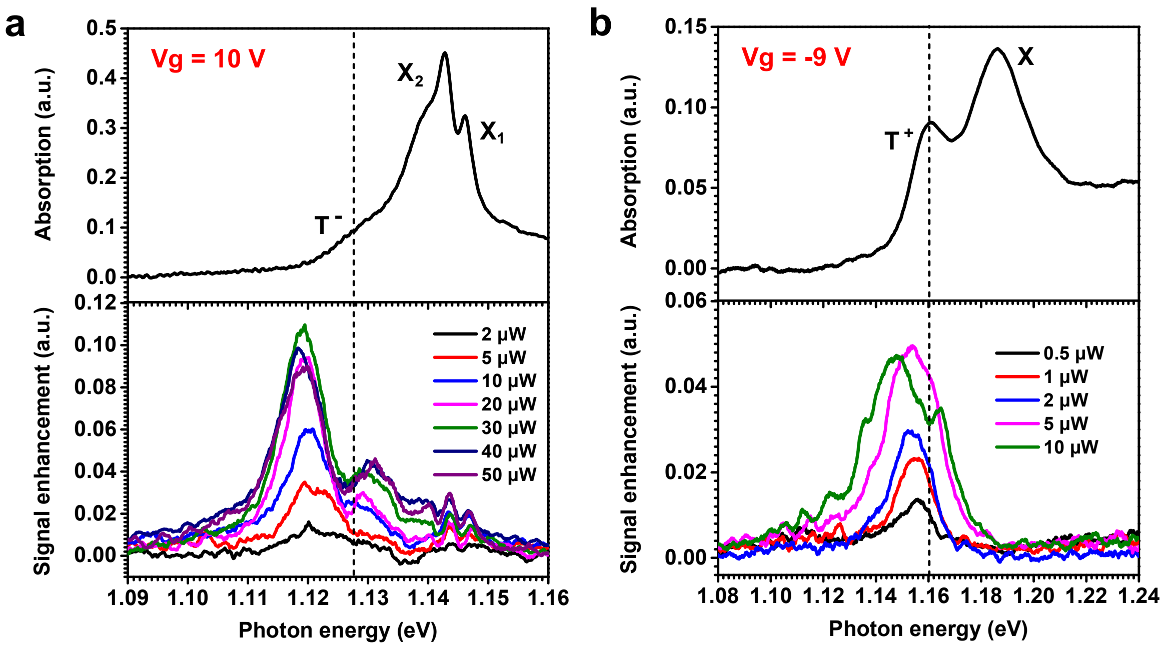

Figure S7 | Absorption and signal enhancement results for **(a)** bilayer and **(b)** monolayer $MoTe_2$ samples, corresponding to Fig. 2 in the main text.

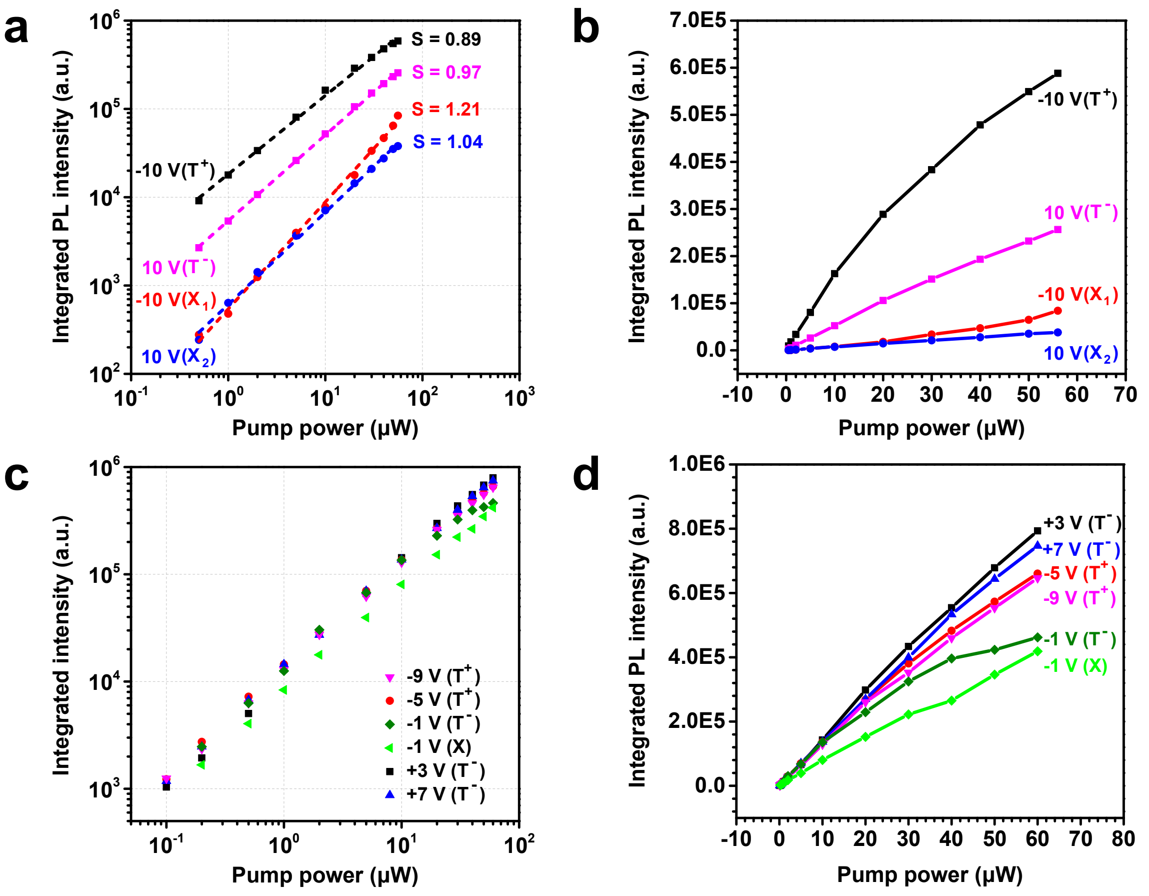

Figure S8 | Log-log plot **(a)** and linear plot **(b)** of integrated PL intensity measured at 4 K and gate voltages of +/- 10 V versus pump excitation for the same bilayer sample as presented in Fig. 2 (a,b,c) in the main text. **c. & d.** Similar plots for the same monolayer sample as in Fig. 2 (d,e,f) in the main text.

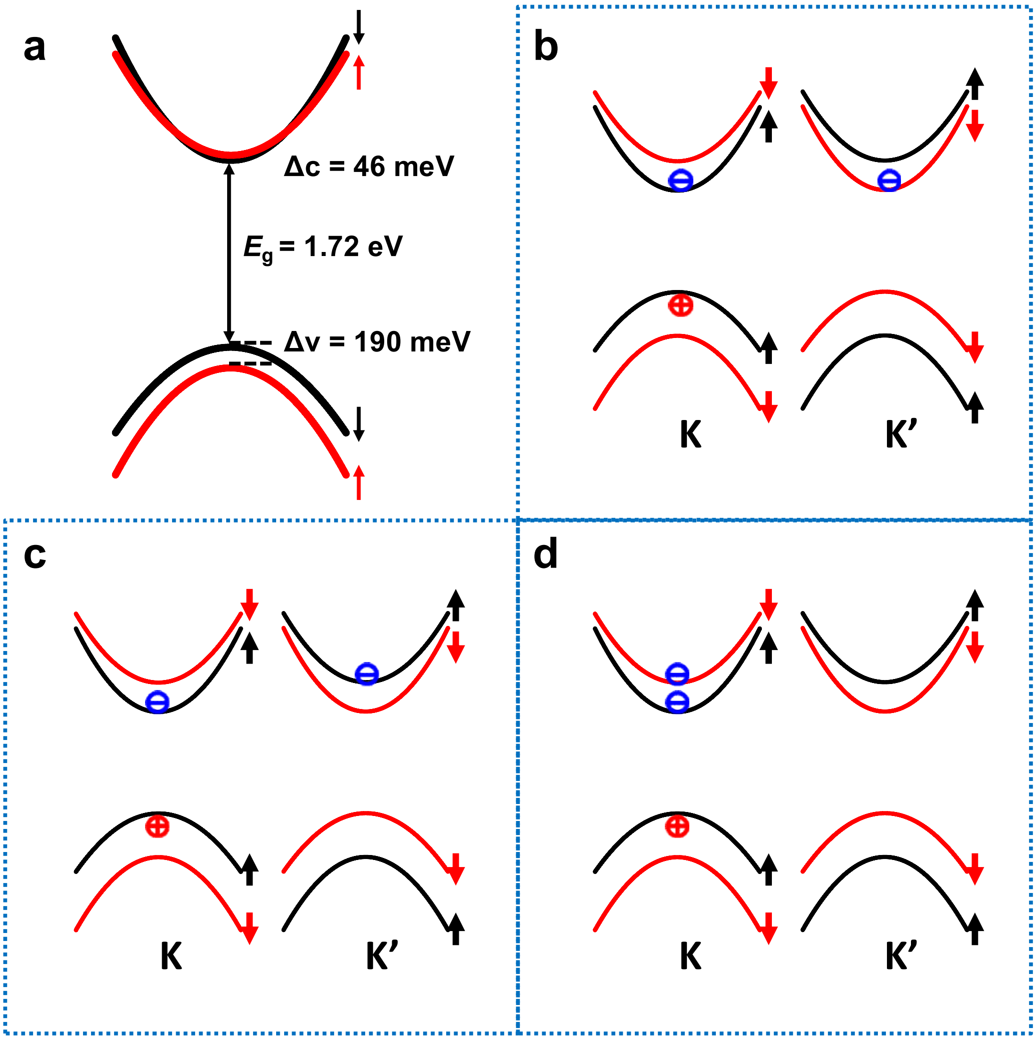

Figure S9 | a. Schematic of the electronic band structure of $MoTe_2$ for the K valley. Δc and Δv are the splittings of the conduction band and valance band, respectively. The arrows indicate the spin directions. Schematic of negative trion compositions, including inter-valley trions (**b & c**) and an intra-valley trion (**d**). Energies are not to scale for clarity.

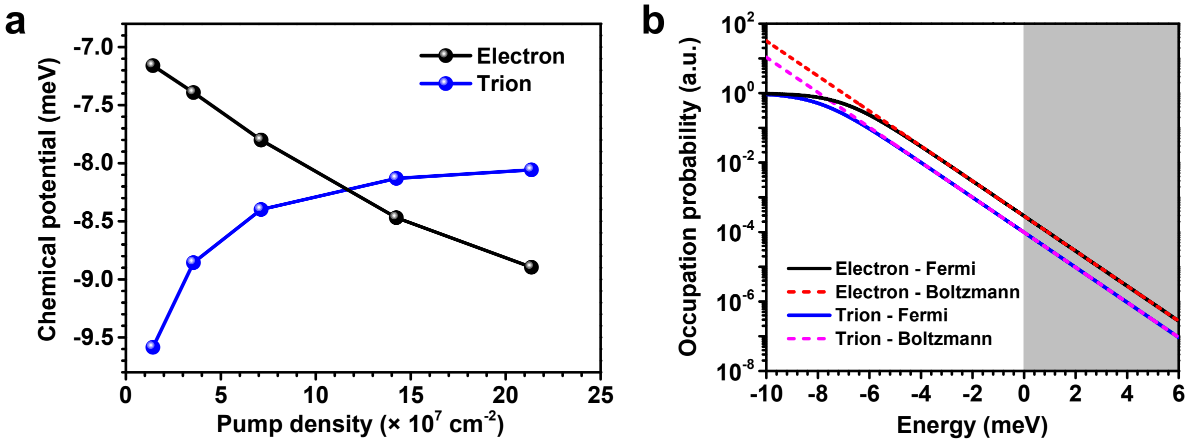

Figure S10 | a. Chemical potential for both electrons and trions versus pump density. **b.** Occupation probability of both electrons and trions at T = 10 K for a carrier density of ~ 7.2×10^7 cm^{-2}. The horizontal axis "0" refers to the conduction band minimum. The shaded region indicates the positive energy region, where carriers are populated with the 2D density of states and the results for the two distribution functions are almost identical.

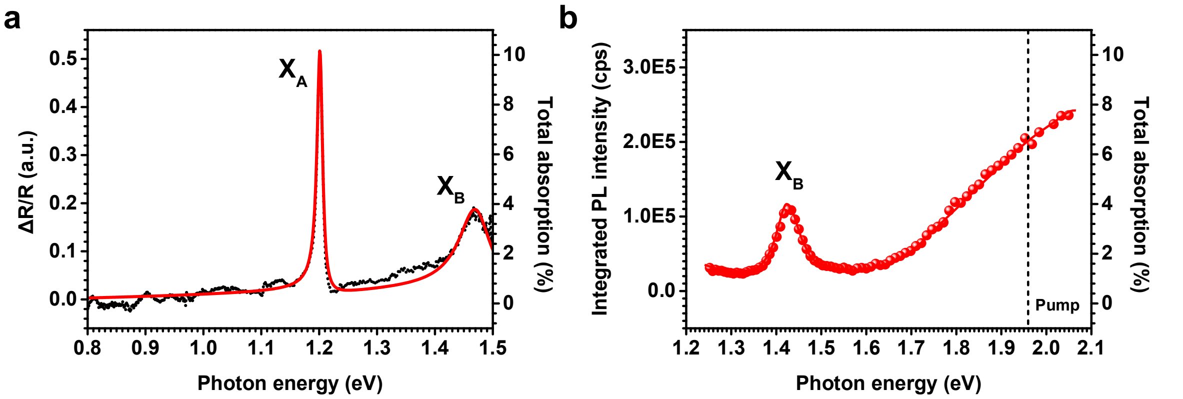

Figure S11 | a. Reflection spectrum of $MoTe_2$ measured on top of a quartz substrate, showing A and B excitons. **b.** PLE measurement results; the absorption at the pump laser energy was determined to be 7 %.

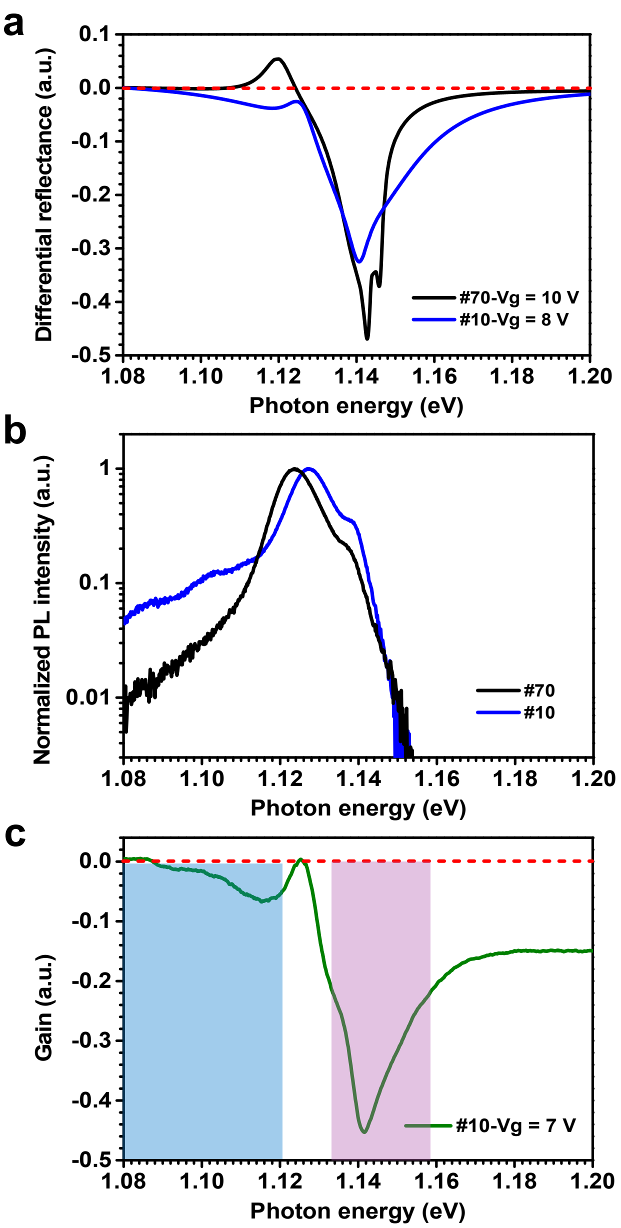

Figure S12 | a. Modelled differential reflectance spectra based on the data at the highest pumping level for the samples in Fig. 2 (a-c) at 10 V and Fig. 4 (a) at 8 V. The exciton linewidth was chosen to correspond to that of sample #70 or sample #10. **b.** Comparison of the PL spectra for the two samples. **c.** Gain spectrum from Fig. 4 (b-c) for sample #10 at 7 V to illustrate the roles of absorptive features on both sides of the trion gain peak.

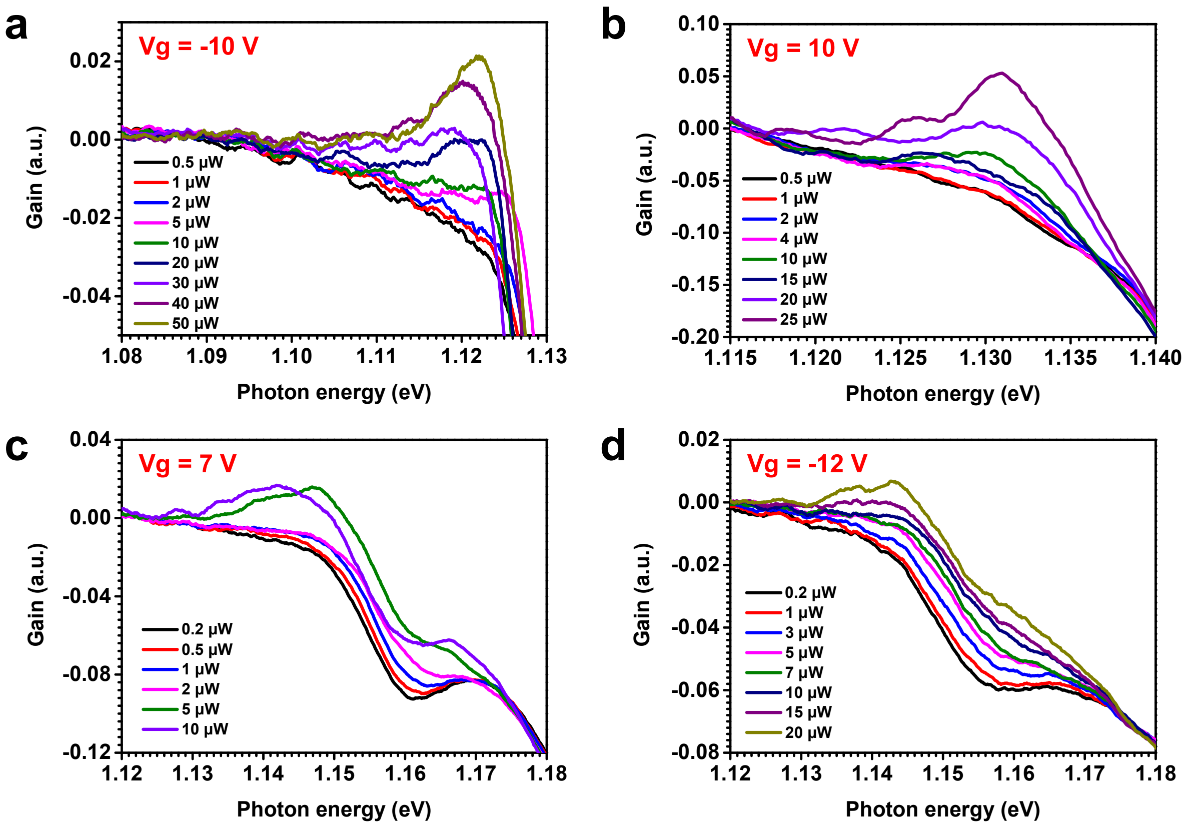

Figure S13 | Gain spectra evolution with increasing pumping level for **(a)** the same bilayer device as in Fig. 2 (a,b,c) in the main text at a gate voltage of -10 V, **(b)** another bilayer device with a similar device structure at 10 V, **(c)** the same monolayer device as in Fig. 2 (d,e,f) in the main text at a gate voltage of 7 V, and **(d)** the same monolayer device as in **(c)** after storage in ambient conditions for three weeks.
